# Downregulation of CPA4 inhibits non small–cell lung cancer growth by suppressing the AKT/c‐MYC pathway

**DOI:** 10.1002/mc.23095

**Published:** 2019-08-09

**Authors:** Yangyang Fu, Lihuang Su, Mengsi Cai, Boyang Yao, Sisi Xiao, Qinlian He, Le Xu, Lehe Yang, Chengguang Zhao, Tingting Wan, Lianyou Shao, Liangxing Wang, Xiaoying Huang

**Affiliations:** ^1^ Division of Pulmonary Medicine, Key Laboratory of Heart and Lung The First Affiliated Hospital of Wenzhou Medical University Wenzhou Zhejiang China; ^2^ Chemical Biology Research Center, School of Pharmaceutical Sciences Wenzhou Medical University Wenzhou Zhejiang China

**Keywords:** apoptosis, carboxypeptidase A4, cell cycle, lung cancer, proliferation

## Abstract

Carboxypeptidase A4 (CPA4) is a member of the metallocarboxypeptidase family. A previous study indicated that CPA4 may participate in the modulation of peptide hormone activity and hormone‐regulated tissue growth and differentiation. However, the role of CPA4 in lung tumorigenesis remains unclear. Our study revealed that CPA4 expression was higher in both lung cancer cells and tumor tissues. We performed 3‐(4,5‐dimethylthiazol‐2‐yl)‐2,5‐diphenyl tetrazolium bromide assays, colony‐formation assays, and Cellomics ArrayScan Infinity analysis to demonstrate that CPA4 knockdown inhibited non small–cell lung cancer (NSCLC) cell proliferation. Conversely, ectopic expression of CPA4 enhanced lung cancer cell proliferation. Consistent with these observations, we generated xenograft tumor models to confirm that CPA4 downregulation suppressed NSCLC cell growth. Mechanistically, we revealed that CPA4 downregulation may induce apoptosis and G1‐S arrest by suppressing the protein kinase B/c‐MYC pathway. These results suggest that CPA4 has an oncogenic effect on lung cancer growth. Taken together, we identified a novel gene in lung cancer that might provide a basis for new therapeutic targets.

**Abbreviations**
APC: allophycocyanin
Bcl‐2: B‐cell lymphoma‐2
CDK4: cyclin‐dependent kinase 4
CPA4: carboxypeptidase A4
EGFR: epidermal growth factor receptor
FBS: fetal bovine serum
MCP: metallocarboxypeptidase
MTT: methylthiazolyldiphenyl‐tetrazolium bromide
NSCLC: non small–cell lung cancer
PBS: phosphate‐buffered saline
PI3K: phosphatidylinositol 3‐kinase
qRT‐PCR: quantitative real‐time polymerase chain reaction
shRNA: short hairpin RNA
TCGA: The Cancer Genome Atlas
TUNEL: terminal deoxynucleotidyl transferase‐mediated dUTP‐digoxigenin nick end labeling

## 1 INTRODUCTION

Lung cancer is the most common cancer and a leading cause of cancer‐related mortality worldwide, accounting for 1.6 million deaths per year.^1^ Non small–cell lung cancer (NSCLC) accounts for 83% of all lung cancer cases, and most people are diagnosed during the late stage. Despite the great advancements that have been achieved in lung cancer therapies, the 5‐year survival rate is still poor.^2^ Hence, identifying novel regulators that participate in lung cancer growth is essential.

Carboxypeptidase A4 (CPA4) is a member of the metallocarboxypeptidase (MCP) family.^3^, ^4^ MCPs are zinc‐dependent enzymes that catalyze the C‐terminus of peptide and protein substrates that functions in digestion and the selective biosynthesis of neuroendocrine peptides.^5^, ^6^ CPA4 was previously shown to play a crucial role in cell growth and differentiation by modulating or inactivating peptide hormone activity resulting from the cleavage of the COOH‐terminus.^4^ Meanwhile, CPA4 is a maternally imprinted gene that is located in a region of chromosome 7q32 that is speculated to be associated with prostate cancer aggressiveness.^7^, ^8^, ^9^ Ross et al^10^ demonstrated that a nonsynonymous coding single‐nucleotide polymorphism in CPA4 correlates with an increased risk of aggressive prostate cancer among younger men (<66 years). Moreover, several studies have shown that CPA4 expression is increased in multiple cancer tissues, including tissues from patients with pancreatic cancer, gastric cancer, esophageal squamous cell carcinoma, and lung cancer, and may serve as a potential diagnostic and prognostic marker.^11^, ^12^, ^13^, ^14^ These findings suggest that CPA4 may play a crucial role in cancers; however, the role of CPA4 in lung tumorigenesis remains poorly understood.

In this study, we investigated the function of CPA4 in lung tumorigenesis. Our work revealed that CPA4 was overexpressed in most human lung cancer tissues, and decreasing CPA4 inhibited lung cancer growth by inducing apoptosis and G1‐S arrest. Mechanistically, we showed that CPA4 activated the protein kinase B(AKT)/c‐MYC pathway to promote lung tumorigenesis.

## 2 MATERIALS AND METHODS

### 2.1 Reagents

Roswell Park Memorial Institute 1640 medium was purchased from Thermo Fisher Scientific (Waltham, MA). F‐12K medium was purchased from Sigma‐Aldrich (St Louis, MO). The rabbit antibody against c‐MYC was purchased from Abcam (Cambridge, UK). The rabbit antibody against CPA4 was purchased from Genetex (Irvine, CA). The rabbit antibodies against α‐tubulin, β‐actin, P27, cyclin‐dependent kinase 4 (CDK4), B‐cell lymphoma‐2 (Bcl‐2), caspase 9, caspase 3, p‐AKT, and AKT and the mouse antibody against flag were purchased from Cell Signaling Technology (Beverly, MA). The mouse antibody against Cyclin D1 was purchased from Santa Cruz Biotechnology (Santa Cruz, MO).

### 2.2 Tissue samples and cell culture

Fresh lung cancer samples and matched adjacent tissues were collected from the First Affiliated Hospital of Wenzhou Medical University with the approval of the Ethics Committee of the First Affiliated Hospital of Wenzhou Medical University and written informed consent of all the participants. The cell lines Beas‐2B, NCI‐H1299, A549, NCI‐H1688, and NCI‐H1975 were obtained from the Cell Bank of the Chinese Academy of Sciences (Shanghai, China). Routine cell cultures were maintained in medium containing 10% fetal bovine serum and 1% penicillin/streptomycin in a humidified 5% CO_2_ atmosphere at 37°C.

### 2.3 Quantitative real‐time polymerase chain reaction

The total RNA was extracted from the tumor tissues and lung cancer cells using TRIzol reagent (Invitrogen, Carlsbad, CA). Complementary DNA was synthesized with murine leukemia virus‐reverse transcriptase (Invitrogen), and each reaction was performed in triplicate using SYBR Green (Tiangen, Beijing, China) according to the manufacturer's instructions using an ABI7300 real‐time detection system (Applied Biosystems). Glyceraldehyde 3‐phosphate dehydrogenase was used as an internal control. The program was as follows: 95°C for 2 minutes and 40 cycles of 95°C for 15 seconds, 60°C for 30 seconds and 68°C for 30 seconds. A dissociation curve was generated. The fold change in expression was calculated by the formula $2^{-\Delta \Delta C_{t}}$. The primers used in the experiments are listed in Table S1. The quantitative real‐time polymerase chain reaction (qRT‐PCR) data were calculated from triplicate reactions.

### 2.4 Immunohistochemical analysis of a tissue array and mouse tumor tissues

Tissue microarrays containing 131 lung cancer tissues and corresponding nontumor tissues were purchased from Alenabio (Xi’an, China). Mouse tumor tissues were embedded in paraffin for examination. Four‐micrometer‐thick sections were cut and mounted on glass. After deparaffinization and rehydration, the sections were stained with hematoxylin and eosin and analyzed by immunohistochemistry. For the immunohistochemistry, the sections were antigen‐retrieved, washed, and treated with peroxidase (Santa Cruz Biotechnology) for 5 minutes. Each slide was blocked with normal donkey serum for 20 minutes. Then, the slides were incubated with an anti‐CPA4 antibody (1:100; Abcam), and mouse tumor tissues were incubated with an anti‐Ki67 antibody (1:100; Abcam). After washing twice with phosphate‐buffered saline (PBS), the slides were hybridized with a secondary antibody, followed by incubation with a peroxidase‐streptavidin reagent. Then, the color was developed using a diaminobenzidine substrate, and the sections were counterstained with hematoxylin. The results were assessed by two independent pathologists. The following criteria were used to quantify the protein expression level of CPA4: the CPA4 staining intensity was classified as no (score = 0), weak (score = 1), moderate (score = 2), or strong (score = 3) staining; and the positive percentage of CPA4 cells was scaled as 0% (score = 0), 1% to 25% (score = 1), 26% to 50% (score = 2), 51% to 75% (score = 3), or 76% to 100% (score = 4). The values of the staining intensity were multiplied by the percentage to obtain a single CPA4 score for each sample. The mean score of tumors vs adjacent tissues was compared by the Student *t* test (two‐tailed).

### 2.5 Terminal deoxynucleotidyl transferase‐mediated dUTP‐digoxigenin nick end labeling assay

Cell apoptosis was determined using terminal deoxynucleotidyl transferase‐mediated dUTP‐digoxigenin nick end labeling (TUNEL) assay (Promega, Madison, WI). Briefly, paraffin‐embedded tissues were fixed in 4% paraformaldehyde solution for 15 minutes at room temperature after deparaffinization and rehydration. The sections were permeabilized for 15 minutes using proteinase K, followed by incubation with recombinant terminal deoxynucleotidyl transferase reaction mix at 37°C for 60 minutes in a humidified chamber. The slides were immersed in 0.3% hydrogen peroxide in PBS for 5 minutes. Next, the slides were incubated with streptavidin horseradish peroxidase solution for 30 minutes and stained with 3,3′‐diaminobenzidine solution until there was a light brown background.

### 2.6 3‐(4,5‐Dimethylthiazol‐2‐yl)‐2,5‐diphenyl tetrazolium bromide assay

Cells were seeded into 96‐well plates at 2000 cells/well in triplicates and incubated for the indicated times. 3‐(4,5‐Dimethylthiazol‐2‐yl)‐2,5‐diphenyl tetrazolium bromide (MTT; Sigma‐Aldrich) solution was added to the wells, and the samples were cultured for 4 to 6 hours at 37°C. The medium was removed, and dimethyl sulfoxide was added to dissolve the formazan product. The absorbance was determined at 490 nm with a microplate reader (Tecan Infinite, Switzerland).

### 2.7 Colony‐formation assay

Cells were seed into six‐well plates at 1000 cells/well and cultured for 10 days. At the assay endpoint, the cells were washed gently with PBS, fixed with prechilled methanol for 10 minutes and stained with crystal violet for 15 minutes. Stained colonies with more than 50 cells were considered colony‐forming units. The experiment was performed in triplicate wells in three independent experiments.

### 2.8 Apoptosis analysis

Apoptosis was analyzed using an Annexin V‐APC Apoptosis Detection Kit (BD Biosciences, San Jose, CA) according to the manufacturer's instructions. After a 96‐hour transduction with short hairpin RNAs (shRNAs), the cells were subjected to cell apoptosis analysis. Briefly, 5 × 10^5^ cells were harvested by centrifugation at 700 rpm for 5 minutes. The cell pellets were washed with prechilled PBS. Then, the cells were resuspended with 200 µL of 1X binding buffer containing 5 µL of Annexin V‐APC and 5 µL of 7‐aminoactinomycin D and incubated for 15 minutes in the dark at room temperature. After the incubation, 400 µL of 1X binding buffer were added to the cells and then 1 × 10^4^ cells were measured by a flow cytometer (BD FACS Calibur, San Jose, CA). The experiment was performed in triplicate.

### 2.9 Cell cycle analysis

Flow cytometry was used to analyze the cell cycle according to the manufacturer's instructions. After a 96‐hour transduction with shRNAs, the cells were subjected to cell cycle analysis. Briefly, cells (1 × 10^6^) were harvested by centrifugation at 700 rpm for 5 minutes. Then, cells were washed twice with PBS and fixed with ice‐cold 70% ethanol overnight at 4°C. The cell pellets were washed with PBS, followed by resuspension in 500 mL of PBS containing 50 mg/mL propidium iodide, 0.1 mg/mL RNase A, and 0.05% Triton X‐100, and incubated for 15 minutes in the dark at 4°C. Cell cycle distribution was determined using a flow cytometer (Millipore, Boston, MA). The experiment was performed in triplicate.

### 2.10 Cellomics ArrayScan infinity

Cell proliferation was evaluated using a Cellomics ArrayScan Infinity system (Nexcelom, MA) to quantify the number of fluorescent cells. Cells were infected with the lentiviral particles for 72 hours. Then, cells were seeded into 96‐well plates at 2000 cells/well in triplicates and assessed for the indicated durations.

### 2.11 Western blot analysis

Cells were washed and lysed for 15 minutes on ice using lysis buffer supplemented with a protease/phosphatase inhibitor. After a 20‐minute incubation on ice, the lysate was centrifuged at 12 000 rpm for 15 minutes at 4°C. Proteins were resolved by sodium dodecyl sulfate‐polyacrylamide gel electrophoresis and transferred to polyvinylidene difluoride membranes (Bio‐Rad, San Francisco, CA). The membranes were blocked with 5% nonfat milk (Bio‐Rad) in TBST for 2 hours at room temperature, followed by incubation with primary antibodies (anti‐CPA4 diluted 1:1000; anti‐P27 diluted 1:3000; anti‐CDK4 diluted 1:3000; anti‐Cyclin D1 diluted 1:2000; anti‐Bcl‐2 diluted 1:500; anti‐caspase‐9 diluted 1:1000; anti‐caspase‐3 diluted 1:1000; anti‐p‐AKT diluted 1:2000; anti‐AKT diluted 1:3000; anti‐flag diluted 1:3000; anti‐α‐tubulin diluted 1:3000; and anti‐β‐actin diluted 1:3000) overnight at 4°C. Subsequently, the membranes were incubated with secondary antibodies for 2 hours at room temperature. Protein bands were visualized with enhanced chemiluminescence detection reagent (ECL‐Plus, Amersham, Sweden).

### 2.12 Small interfering RNA transfection

The small interfering RNA (siRNA) targeting human c‐MYC was purchased from GenePharma (Shanghai, China). The specific sequence was 5′‐GAGGAUAUCUGGAAGAAAUTT‐3′. At 48 hours after transduction with CPA4‐flag lentivirus, the cells were transfected with 50 nM siRNA using Lipofectamine 2000 (Invitrogen) for 48 hours. Subsequently, the cells were subjected to Western blot analysis with the indicated antibodies.

### 2.13 Lentivirus transduction

The GV143‐CPA4‐flag plasmid and GV115‐CPA4 shRNA were purchased from GeneChem (Shanghai, China). The transduction assay was performed according to the manufacturer's protocols. The specific sequences of shRNAs were as follows: shCPA4 1#: 5′‐GTATGACAACGGCATCAAA‐3′; shCPA4 2#: 5′‐ACCGGCCGATGTATGTACTCCACGAGAUGGACAACAUUTT‐3′. Cells were infected with the lentiviral particles for 72 to 96 hours, and the cells were then subjected to qRT‐PCR analysis, flow cytometry analysis, cell proliferation assays, and Western blot analysis with the indicated antibodies.

### 2.14 Affymetrix gene expression array

Affymetrix gene expression assays were performed according to the manufacturer's protocols (Affymetrix Inc, San Francisco, CA). In brief, the quality control of total RNA from cells was performed with an Agilent 2100 instrument. Amplified RNA was obtained using a GeneChip 3′ IVT Express Kit. The amplified RNA was purified and fragmented, followed by hybridization at 45°C for 16 hours. The GeneChip was washed and stained with GeneChip Fluidics Station 450. The data collection was performed using a GeneChip Scanner3000. The raw data were analyzed with Ingenuity Pathway Analysis software (http://www.ingenuity.com). The cut‐off criteria for differentially expressed genes were an absolute fold change more than 2.0 and a *P* < .05. The significance was evaluated by the Student *t* test.

### 2.15 Xenograft model

Athymic BALB/c nude mice (4‐6‐weeks old) were purchased from the Vital River Experimental Animal Center (Beijing, China). H1299‐shCtrl and H1299‐shCPA4 1# cells (1 × 10^7^ cells) were suspended in 100 µL of PBS and subcutaneously injected into mouse axillas. The tumor volume was measured every 3 to 5 days with a calliper, and the volume was calculated as *v* = length × width^2^ × 0.5326. All animal experiments were approved by the Institutional Animal Care and Use Committee of Wenzhou Medical University and performed after the guidelines and regulations.

#### 2.15.1 Cancer patient data analysis

CPA4 messenger RNA (mRNA) expression in NSCLC tissues and normal tissues from The Cancer Genome Atlas (TCGA) datasets was analyzed using UALCAN cancer database (http://ualcan.path.uab.edu/analysis.html). Kaplan‐Meier survival analysis was performed using Kaplan‐Meier Plotter (http://kmplot.com). The patients samples were divided into two groups to analyze the prognostic value of CPA4. Then, the two cohorts were analyzed by Kaplan‐Meier survival plot. The cut‐off level for the categories was the median; the significance was evaluated by the logrank test.

### 2.16 Statistical analysis

All data are shown as the mean ± SD. Unless stated otherwise, statistical analyses were conducted using the Student *t* test. Significance was defined as *P* < .05.

## 3 RESULTS

### 3.1 CPA4 is upregulated in human lung cancer cells and primary tumors, and its expression is associated with poor prognosis

**Figure 1:**
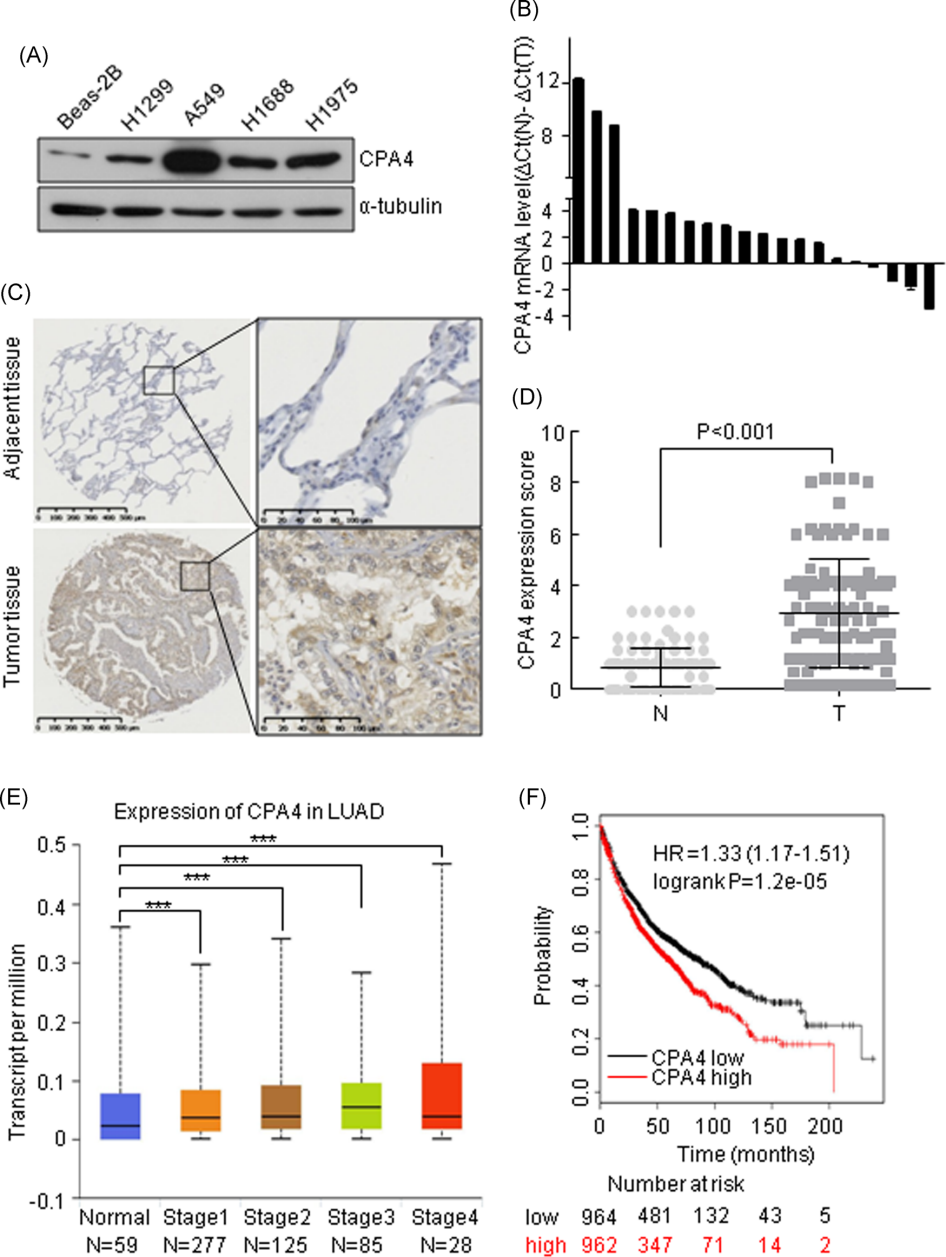
CPA4 expression is upregulated in NSCLC. A, CPA4 expression in lung cancer cell lines and normal cell line Beas‐2B by Western blot analysis. α‐tubulin was used as a control. B, qRT‐PCR analysis of the relative mRNA expression of CPA4 in 20 tumor tissues. CPA4 expression was normalized to GAPDH. The data were calculated from triplicate reactions. Each bar represents the value of ∆∆*C*_t_= ∆*C*_t_(N)−∆*C*_t_(T); N, adjacent tissues; T, lung tumor tissues. C, Immunohistochemical staining of CPA4 in NSCLC tissues and matched nontumor tissues. Scale bar = 100 μm. D, CPA4 expression scores in NSCLC tissues and adjacent tissues. CPA4 expression score is the product of the staining intensity and positive percentage; N, adjacent tissues; T, lung tumor tissues. E, CPA4 mRNA expression in NSCLC tissues and normal tissues from TCGA datasets (***P < .001). F, Kaplan‐Meier survival analysis of CPA4 expression in patients with lung cancer subdivided with two groups. The cut‐off level for the categories was the median. Data are shown as mean ± SD from three independent experiments (***P < .001). CPA4, carboxypeptidase A4; GAPDH, glyceraldehyde 3‐phosphate dehydrogenase; LUAD, Lung adenocarcinoma; mRNA, messenger RNA; NSCLC, non small–cell lung cancer; qRT‐PCR, quantitative real‐time polymerase chain reaction; TCGA, The Cancer Genome Atlas [Color figure can be viewed at wileyonlinelibrary.com]

We first examined CPA4 expression in lung cancer cells and lung normal epithelial cells. The result showed that CPA4 protein level was significantly overexpressed in lung cancer cells compared with that in lung normal epithelial cell Beas‐2B (Figure 1A). Then, we performed qRT‐PCR to evaluate CPA4 gene expression in 20 tumor tissues and corresponding nontumor tissues. We found that CPA4 mRNA was significantly upregulated in 14 tumor tissues (Figure 1B). To confirm this result, we expanded the number of lung cancer tissues to evaluate CPA4 protein expression by immunohistochemistry. We multiplied the staining intensity by the percentage to obtain a score for the quantification of CPA4 protein expression. The results showed that CPA4 was increased in 93 of the 131 (71%) tumor tissues compared with that in the adjacent tissues (Figure 1C and 1D). Meanwhile, we analyzed TCGA datasets for CPA4 expression. The results indicated that CPA4 expression was higher in NSCLC tissues compared with that in normal tissues (Figure 1E). Further, the Kaplan‐Meier survival curve showed that high CPA4 expression was correlated with poor prognosis (Figure 1F). These results indicate that CPA4 may serve as a novel diagnostic marker of NSCLC.

### 3.2 CPA4 promotes lung cancer cell growth

**Figure 2:**
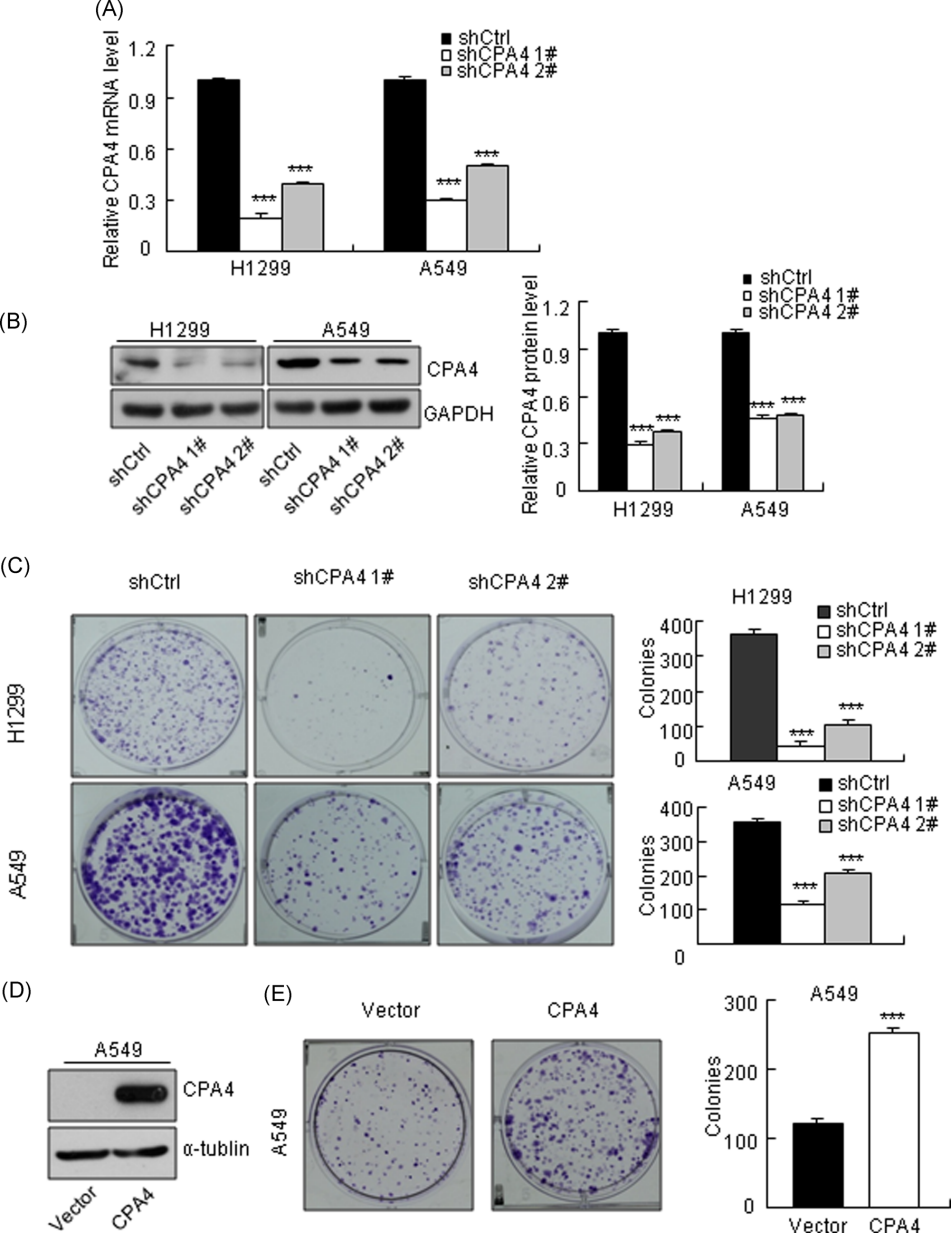
CPA4 promotes the clonogenic ability of lung cancer cells. A, qRT‐PCR results of CPA4 expression in shRNA‐transduced cells. CPA4 expression was normalized to GAPDH. After a 72‐hour transduction with shRNAs, the cells were subjected to qRT‐PCR. The data were calculated from triplicates (***P < .001). B, Western blot analysis of CPA4 protein expression in lung cancer cells after CPA4 knockdown. GAPDH was used as a control. After a 72‐hour transduction with shRNAs, the cells were subjected to Western blot analysis. The data were calculated from triplicates (***P < .001). C, Representative results of colony‐formation assays in lung cancer cells. The numbers of colonies are shown in the right panel. After a 72‐hour transduction with shRNAs, cells were seeded into six‐well plates at 1000 cells/well and cultured for 10 days. Stained colonies with more than 50 cells were considered colony‐forming units. The experiment was performed in triplicate wells in three independent experiments (***P < .001). D, Western blot analysis of CPA4 protein expression in lung cancer cells following ectopic expression of flag‐tagged CPA4. Flag‐tagged antibody was exploited to detect CPA4 expression. α‐Tubulin was used as a control. After a 72‐hour transduction with CPA4‐flag lentivirus, the cells were subjected to Western blot analysis. E, Representative results of colony‐formation assay in CPA4‐overexpressing A549 cells. The quantitative numbers of colonies are shown in the right panel. After a 72‐hour transduction with CPA4‐flag lentivirus, cells were seeded into six‐well plates at 1000 cells/well and cultured for 10 days. Stained colonies with more than 50 cells were considered colony‐forming units. The experiment was performed in triplicate wells in three independent experiments (***P < .001 vs control). Data are shown as mean ± SD from three independent experiments (***P < .001). CPA4, carboxypeptidase A4; GAPDH, glyceraldehyde 3‐phosphate dehydrogenase; mRNA, messenger RNA; qRT‐PCR, quantitative real‐time polymerase chain reaction; shRNA, short hairpin RNA [Color figure can be viewed at wileyonlinelibrary.com]

**Figure 3:**
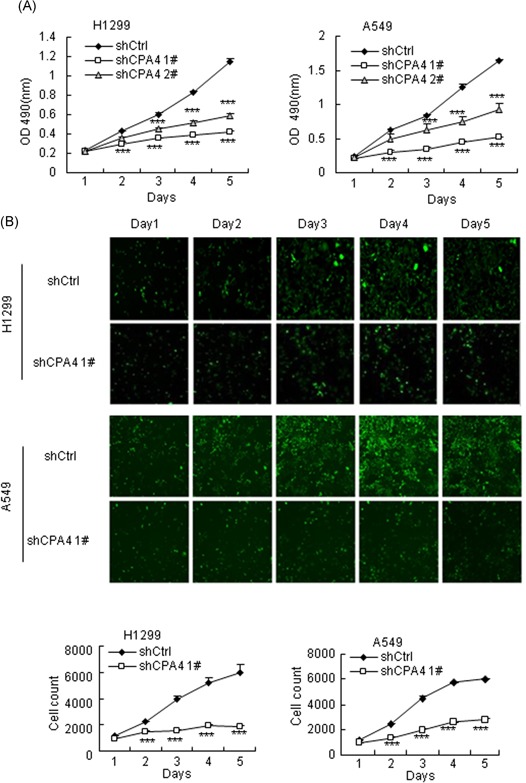
Knockdown of CPA4 inhibits lung cancer cells growth. A, Cell viability of lung cancer cells following CPA4 knockdown and corresponding control cells. After a 72‐hour transduction with shRNAs, cells were seeded into 96‐well plates at 2000 cells/well and determined for the indicated times. The data were calculated in triplicate (***P < .001) vs control. B, Cell proliferation was determined by Cellomics ArrayScan Infinity instruments (×40). After a 72‐hour transduction with shRNAs, cells were seeded into 96‐well plates at 2000 cells/well and determined for the indicated times. The data were calculated in triplicate (***P < .001 vs control). Data are shown as mean ± SD from three independent experiments (***P < .001). CPA4, carboxypeptidase A4; shRNA, short hairpin RNA [Color figure can be viewed at wileyonlinelibrary.com]

To explore the function of CPA4 in lung cancer, we generated CPA4 knockdown cells by transducing *CPA4* shRNAs into H1299 and A549 cells. The effects of CPA4 knockdown were confirmed by both qRT‐PCR and Western blot analysis (Figure 2A and 2B). Colony‐formation assays revealed that CPA4 depletion (shCPA4) dramatically reduced the clonogenicity of lung cancer cells compared with nontargeted (shCtrl) cells (Figure 2C). In contrast, CPA4 overexpression in A549 cells markedly enhanced the clonogenic ability compared with vector control cells (Figure 2D and 2E). Moreover, MTT assays showed that CPA4 downregulation resulted in a significant decrease in cell proliferation compared with that in nontargeted cells in both H1299 and A549 cells (Figure 3A). We further performed proliferation analysis with Cellomics ArrayScan Infinity in H1299 and A549 cells. Similarly, the results indicated that decreased CPA4 led to an inhibition of lung cancer cell growth (Figure 3B). Taken together, these results indicate that CPA4 promotes lung cancer cells growth.

### 3.3 Knockdown of CPA4 induces G1‐S arrest and apoptosis

**Figure 4:**
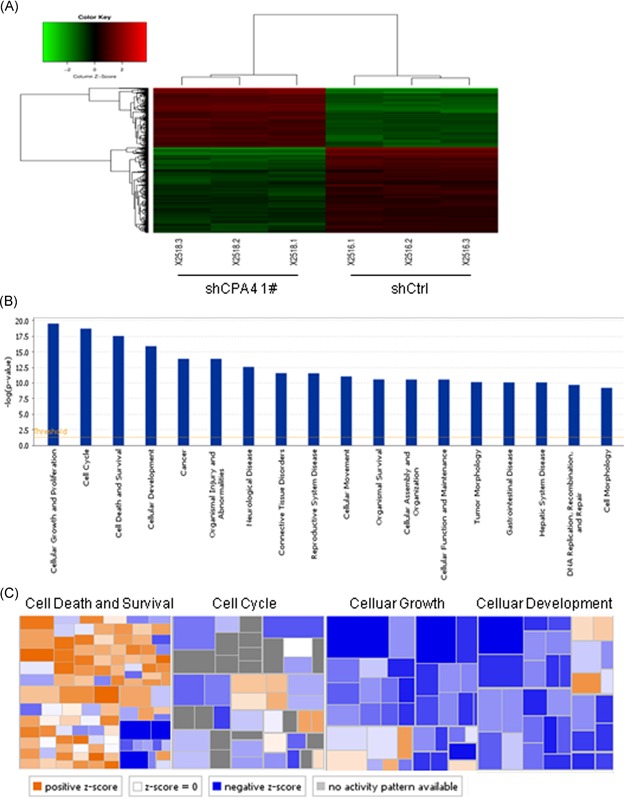
Bioinformatic analysis of CPA4‐regulated functions. A, Heatmap showing the differentially expressed genes measured by Affymetrix gene expression assays. The cut‐off criteria were an absolute fold change ≥2 and *P* < .05. Red represents upregulated genes; green represents downregulated genes; black represents no change. B, Histogram showing the functional classification of CPA4‐regulated genes. Differentially expressed genes were grouped and statistically analyzed using IPA. C, Heatmap showing the functional classification of CPA4‐regulated genes. Differentially expressed genes were grouped and statistically analyzed using IPA. Orange represents a *Z* score >2; blue represents a *Z* score <−2; white represents a *Z* score = 0; gray represents no *Z* score; *Z* score >2 indicates that differentially expressed genes activate this function; *Z* score <−2 indicates that differentially expressed genes inhibit this function. CPA4, carboxypeptidase A4; IPA, ingenuity pathway analysis [Color figure can be viewed at wileyonlinelibrary.com]

**Figure 5:**
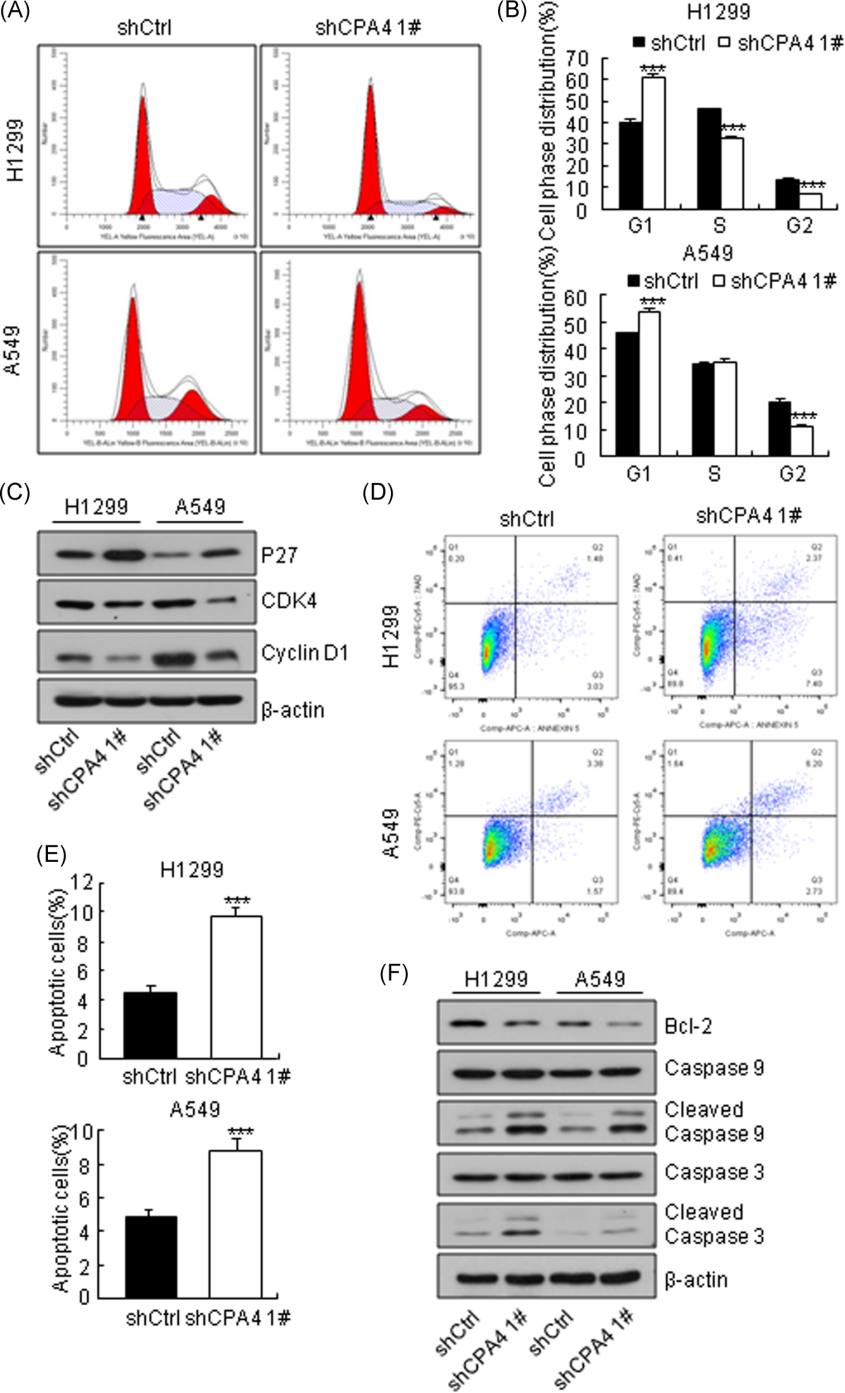
Knockdown of CPA4 results in G1‐S arrest and apoptosis. A, Flow cytometry analysis of cell cycle in shCtrl and shCPA4 1# lung cancer cells. After a 96‐hour transduction with shRNAs, the cells were subjected to cell cycle analysis. The experiment was performed in triplicate. B, Histograms of cell cycle analysis shown in panel A (***P < .001). C, Western blot analysis of cell cycle‐related proteins. β‐Actin was used as a control. After a 96‐hour transduction with shRNAs, the cells were subjected to Western blot analysis with the indicated antibodies. The data are representative of three independent experiments. D, Apoptosis in shCtrl and shCPA4 1# lung cancer cells as determined by flow cytometry. After a 96‐hour transduction with shRNAs, the cells were subjected to cell apoptosis analysis. The experiment was performed in triplicate. E, Histograms of the apoptosis results were shown in panel C (**P < .001). F, Western blot analysis of apoptosis‐related proteins and β‐actin was used as a control. After a 96‐hour transduction with shRNAs, the cells were subjected to Western blot analysis with the indicated antibodies. Data are shown as mean ± SD from three independent experiments (***P < .001). CDK4, cyclin‐dependent kinase 4; CPA4, carboxypeptidase A4; shRNA, short hairpin RNA [Color figure can be viewed at wileyonlinelibrary.com]

To investigate the mechanism by which CPA4 enhances cell growth, we performed a genome‐wide analysis of gene expression changes following CPA4 knockdown in H1299 cells. The results showed that CPA4 expression was significantly decreased (−1.64‐fold change; *P* = .002) in the CPA4 knockdown cells compared with shCtrl cells (data not shown). Using an absolute fold change of at least 2.0 and *P* < .05, we identified 360 upregulated genes and 460 downregulated genes in the knockdown cells compared with shCtrl cells (Figure 4A; Table S2). Then, we performed functional classification based on 820 genes whose expression levels were altered upon CPA4 knockdown using ingenuity pathway analysis. The results showed that these differentially expressed genes were enriched in various biological functions, including cellular growth, cell cycle, cell death, and cellular development (Figure 4B and 4C). Considering the pivotal role of cell cycle distribution and apoptosis in cell growth, we assessed the effect of CPA4 on cell cycle and apoptosis by flow cytometry. Loss of CPA4 in H1299 cells led to a 20.1% increase in G1 phase and 13.8% and 6.3% decreases in S and G2 phases, respectively. In A549 cells, CPA4 knockdown resulted in an 8.2% increase in the G1 phase and a concomitant decrease in G2 phase (Figure 5A and 5B). In agreement with this result, CPA4 downregulation reduced the expression of Cyclin D1 and CDK4 and elevated the expression of P27 (Figure 5C). Furthermore, CPA4 knockdown elicited a significant increase in the number of apoptotic H1299 cells (9.65 ± 0.67% vs 4.53 ± 0.35%; *P* < .001) and A549 cells (8.77 ± 0.61% vs 4.85 ± 0.6%; *P* < .001) compared with shCtrl cells (Figure 5D and 5E). Concurrently, CPA4 knockdown cells exhibited a decrease of antiapoptosis protein Bcl‐2 and an increase of proapoptosis protein cleaved‐caspase 9 and cleaved‐caspase 3 compared with shCtrl cells (Figure 5F). Together, these results indicate that CPA4 knockdown inhibits lung cancer cells growth by inducing G1‐S arrest and cell apoptosis.

### 3.4 CPA4 promotes lung cancer cells growth via the AKT/c‐MYC pathway

**Figure 6:**
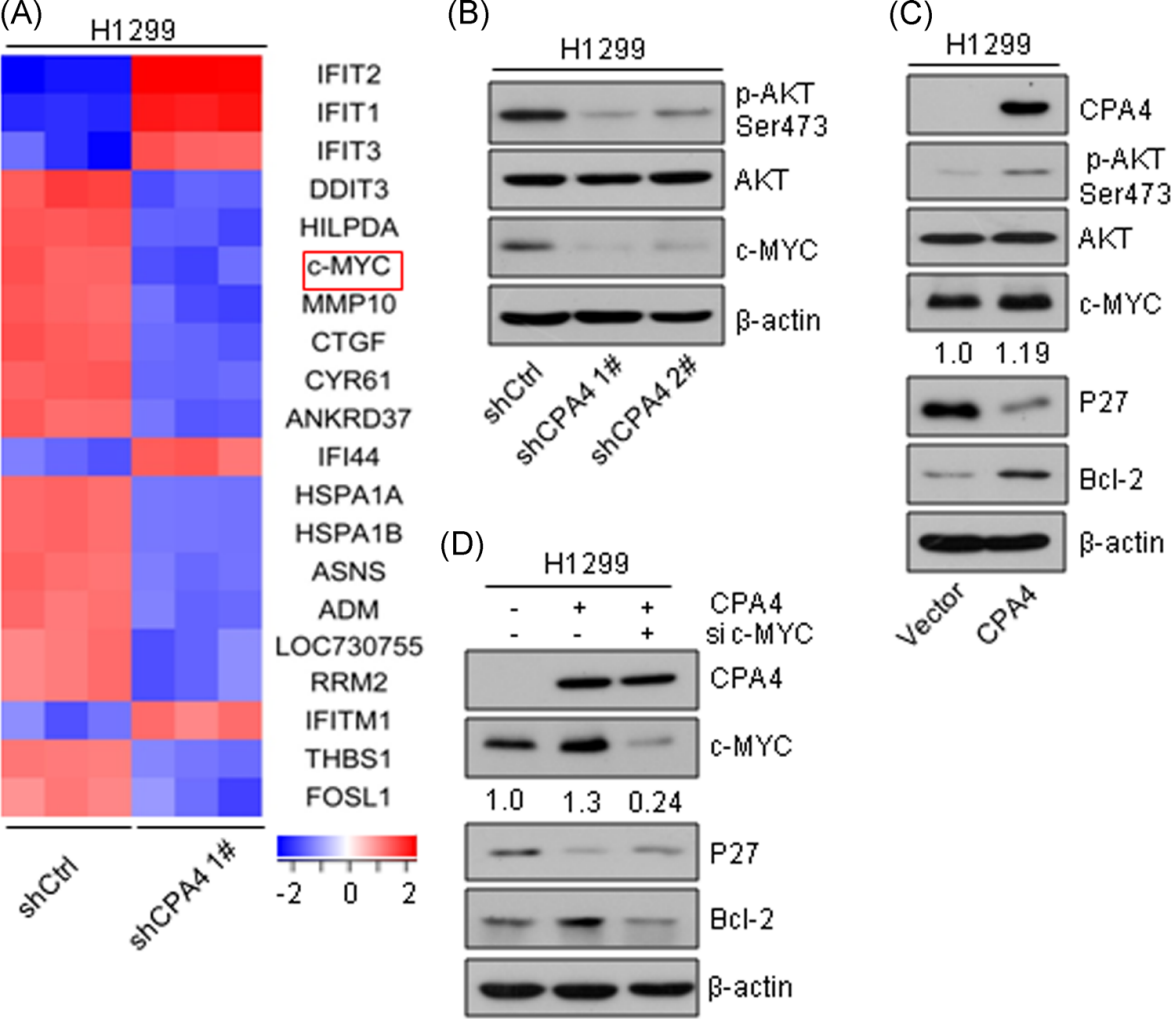
Downregulation of CPA4 suppresses the AKT/c‐MYC pathway to induce apoptosis and G1‐S arrest. A, Heatmap showing the expression level of the top 20 CPA4‐regulated genes (fold change ≥2 and *P* < .05) as determined by Affymetrix gene expression assays. B, Western blot analysis of p‐AKT, AKT, and c‐MYC in shCtrl, and shCPA4 H1299 cells and β‐actin was used as a control. After a 96‐hour transduction with shRNAs, the cells were subjected to Western blot analysis with the indicated antibodies. C, Western blot analysis of p‐AKT, AKT, c‐MYC, P27, and Bcl‐2 in CPA4‐overexpressing H1299 cells. β‐Actin was used as a control. After a 96‐hour transduction with shRNAs, cells were subjected to Western blot analysis with the indicated antibodies. D, Western blot analysis of P27 and Bcl‐2 after depletion of c‐MYC in CPA4‐overexpressing H1299 cells and β‐actin was used as a control. At 48 hours after transduction with CPA4‐flag lentivirus, the cells were transfected with siRNA for 48 hours. Subsequently, the cells were subjected to Western blot analysis with the indicated antibodies. AKT, protein kinase B; Bcl‐2, B‐cell lymphoma‐2; CPA4, carboxypeptidase A4; shRNA, short hairpin RNA; siRNA [Color figure can be viewed at wileyonlinelibrary.com]

To explore the molecular mechanism involved in the regulation of CPA4 depletion‐induced cell cycle arrest and apoptosis, we further ranked the 820 differentially expressed genes from the microarray. Of the top 20 differentially expressed genes, two genes (c‐MYC and RRM2) were related to cell cycle, seven genes (DDIT3, c‐MYC, CYR61, HSPA1A, HSPA1B, ASNS, and FOSL1) were related to cell apoptosis (Figure 6A). These results suggested that c‐MYC may be a potential downstream target mediating the effect of CPA4 knockdown on lung tumorigenesis, which has been reported to play an important role in cell cycle regulation and cell apoptosis.^15^, ^16^ Indeed, Western blot analysis result showed that CPA4 knockdown led to a marked decrease of c‐MYC expression compared with nontargeting cells (Figure 6B). In contrast, ectopic expression of CPA4 resulted in an elevated expression of c‐MYC and P27 and a decreased expression of Bcl‐2 (Figure 6C). To determine whether c‐MYC could be the cause that CPA4 inhibits apoptosis and promotes cell proliferation, we knocked down c‐MYC expression using c‐MYC siRNA in CPA4‐overexpressing cells. The results indicated that depletion of c‐MYC abolished the decrease of P27 and elevation of Bcl‐2 induced by CPA4 overexpression (Figure 6D). Thus, c‐MYC is required for CPA4 to promote lung cancer cell growth. Further, we sought to explore how CPA4 regulates c‐MYC. Studies have reported that the transcription of c‐MYC can be induced via AKT pathway.^17^, ^18^ In addition, MCP subfamily carboxypeptidase E (CPE) has been shown to increase the phosphorylation of AKT.^19^ Thus, we examined whether CPA4 affected the phosphorylation of AKT. The result showed that CPA4 depletion impaired AKT phosphorylation (Figure 6B). Conversely, CPA4 overexpression increased the protein levels of phosphorylated‐AKT (Figure 6C). Taken together, these results suggest that CPA4 enhances lung cancer growth via the AKT/c‐MYC pathway.

### 3.5 Knockdown of CPA4 inhibits subcutaneous tumor growth in nude mice

**Figure 7:**
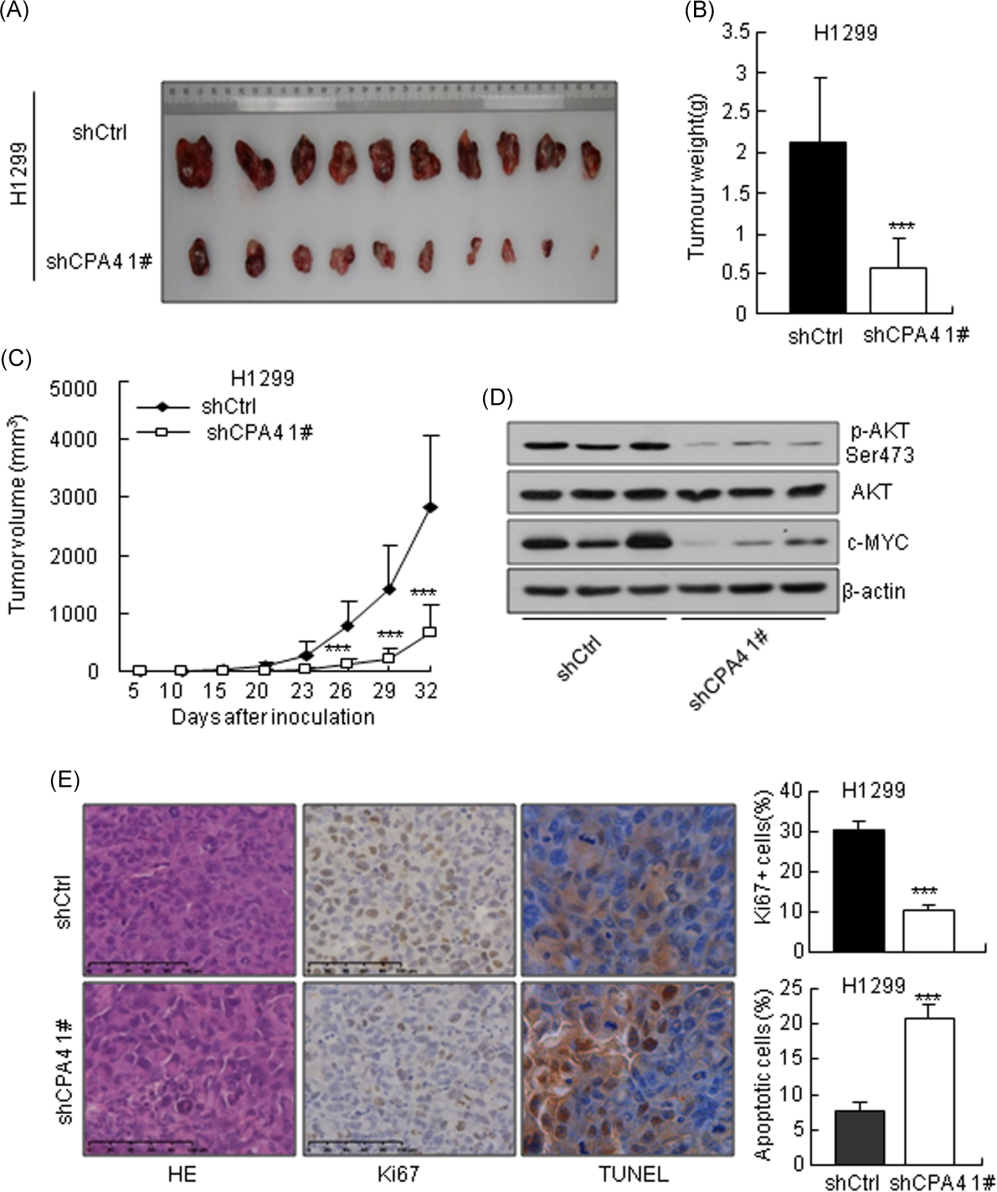
Depletion of CPA4 inhibits lung cancer growth in vivo. A, Image of tumor volume at the termination of the experiment. B, Tumor weight was measured at the end of the experiment (***P < .001). C, Growth curve of subcutaneous tumors formed by H1299 cells following CPA4 knockdown or control shRNA (**P < .01, ***P < .001). D, Western blot analysis of p‐AKT, AKT, and c‐MYC in tumor xenograft tissues. β‐Actin was used as a control. E, Representative micrographs of hematoxylin and eosin histology, immunohistochemistry staining for Ki67 and TUNEL of tumors. The quantitative numbers of Ki67^+^ cells and apoptotic cells are shown in the right panel. The data are representative of three independent experiments. Scale bar = 100 μm (***P < .001). AKT, protein kinase B; CPA4, carboxypeptidase A4; shRNA, short hairpin RNA; TUNEL, terminal deoxynucleotidyl transferase‐mediated dUTP‐digoxigenin nick end labeling [Color figure can be viewed at wileyonlinelibrary.com]

To evaluate the suppressive effect of CPA4 on lung tumor growth in vivo, we generated xenograft tumor models by subcutaneously injecting H1299‐shCPA4 1# and H1299‐shCtrl cells into nude mice. The result showed that CPA4 knockdown inhibited tumor growth in nude mice (Figure 7A). The weight and volume of tumors derived from H1299‐shCPA4 1# cells were significantly reduced compared with H1299‐shCtrl cells (Figure 7B and 7C). Further, we assessed the protein levels of p‐AKT and c‐MYC in tumor xenograft tissues. In accordance with our above results, Western blot analysis revealed that p‐AKT and c‐MYC expression was decreased in the CPA4 knockdown tumor tissues compared with shCtrl tumor tissues (Figure 7D). In addition, fewer proliferative cells were detected in the tumors derived from H1299‐shCPA4 1# cells than that derived form H1299‐shCtrl cells by Ki67 staining (Figure 7E). TUNEL assay results showed a higher level of cell apoptosis in CPA4 knockdown tumor tissues (Figure 7E). Overall, these data indicated that downregulation of CPA4 inhibits lung cancer growth in vivo.

## 4 DISCUSSION

The MCP family is classified as CP A/B and CP N/E according to the amino acid sequence similarities and overall domain structure.^5^ CPA4 belongs to the CP A/B subfamily and shares 54% and 63% homology with CPA1 and CPA2, respectively.^20^ MCP has been reported to be linked to tumorigenesis. CPA7 elicited an inhibitory effect on prostate cancer growth by suppressing STAT3 signaling.^21^ N‐terminally truncated splice variant of CPE promoted osteosarcoma migration and invasion via the Wnt/β‐catenin pathway.^22^ However, whether CPA4 functions in tumorigenesis is still unknown. In this study, we demonstrated that CPA4 depletion suppressed lung cancer growth in vitro and in vivo. Mechanistically, CPA4 knockdown decreased the expression of c‐MYC through the AKT pathway, thus inducing G1‐S arrest and cell apoptosis. Our work indicates that CPA4 plays an oncogenic role in lung cancer.

We identified that CPA4 expression was increased in lung cancer cells and most tumor tissues. This finding is consistent with previous reports that CPA4 was overexpressed in NSCLC, gastric cancer, and pancreatic cancer.^11^, ^12^, ^14^ Further, loss and gain of function experiments demonstrated that CPA4 promoted lung cancer cell growth. Moreover, we established a xenograft tumor model to confirm the above‐mentioned results. Mechanistically, bioinformatics data suggested that CPA4 may play an important role in cell proliferation and cell apoptosis. Indeed, flow cytometry results showed that downregulation of CPA4 induced G1‐S arrest and cell apoptosis. Meanwhile, the alterations of cell cycle and apoptosis‐related proteins confirmed these results. Consistent with our data, MCP subfamily of CPE inhibits H_2_O_2_‐induced cell apoptosis in hippocampal neurons.^19^ These results suggest that CPA4 promotes lung cancer growth though promoting cell proliferation and inhibiting cell apoptosis.

We also explored how CPA4 regulates lung cancer cell proliferation and apoptosis. The gene expression profiles suggested that c‐MYC was a potential candidate target controlled by CPA4. c‐MYC is a transcription factor that is overexpressed in many malignancies. The aberrant expression of c‐MYC promotes tumorigenesis by accelerating cell cycle progression and inhibiting cell apoptosis.^23^, ^24^, ^25^ Our results showed that CPA4 downregulation reduced the expression of c‐MYC. Knockdown of c‐MYC in CPA4‐overexpressing cells blocked the increase of Bcl‐2 and restored the expression of P27, indicating that c‐MYC may be the cause of CPA4 promoting lung cancer growth. In accordance with our findings, N‐terminally truncated CPE has been reported to induce c‐MYC expression to promote osteosarcoma cells migration and invasion.^22^ Thus, these results indicated that CPA4 promotes NSCLC growth via c‐MYC.

We further found that CPA4 knockdown impaired AKT phosphorylation. The AKT pathway participates in multiple cellular processes, including cell proliferation, apoptosis, and cell cycle. Studies have reported that activation of AKT pathway promotes tumorigenicity of breast cancer cells and survival of lymphoid cells via the upregulation of c‐MYC.^17^, ^18^ In addition, the cooperation of activated AKT and c‐MYC induces cell proliferation and transformation.^26^ Our study found that CPA4 promoted cell cycle progression and inhibited cell apoptosis though the AKT/c‐MYC pathway. The activation of AKT pathway is generally considered to be controlled by phosphatidylinositol 3‐kinase (PI3K) and epidermal growth factor receptor (EGFR) activation.^27^ We first performed Western blot analysis to examine whether CPA4 can alter the phosphorylation of EGFR. The result indicated that CPA4 did not affect EGFR activity (data not shown). PI3K is a lipid kinase that comprises of a p85 regulatory subunit and a p110 catalytic subunit. Activation of p85 relieves the inhibition of p110 by p85 and activates the AKT pathway.^28^ Meanwhile, Cheng et at^19^ reported that MCP subfamily of CPE inhibited H_2_O_2_‐induced cell apoptosis through the PI3K/AKT pathway. On the basis of these findings, we propose that CPA4 may be secreted to extracellular domain as an extracellular stimulus to activate p85, thereby enhancing the AKT activity. This hypothesis is still being explored.

Collectively, we found a novel gene in lung cancer that was overexpressed in tumor tissues. We demonstrated that CPA4 knockdown suppressed lung cancer growth in vitro and in vivo. Suppressing CPA4 expression induced G1‐S arrest and cell apoptosis by inhibiting the AKT/c‐MYC pathway. These results indicate that CPA4 may be a potential target for the treatment of NSCLC.

## 5 CONCLUSION

In summary, our work showed that CPA4 downregulation inhibits lung tumorigenesis by regulating the AKT/c‐MYC pathway. This finding might serve as a basis for the treatment of NSCLC.

## ACKNOWLEDGMENTS

We thank the Cardiothoracic Surgery Department of the First Affiliated Hospital of Wenzhou Medical University for kindly providing the tumor tissues.

## CONFLICT OF INTERESTS

The authors declare that there are no conflict of interests.

## DATA ACCESSIBILITY

The datasets used and/or analyzed during the current study are available from the corresponding author on reasonable request.

## Supporting information

Supplementary information

Supplementary information
