# Supplementary material for: Downregulation of CPA4 inhibits non small–cell lung cancer growth by suppressing the AKT/c‐MYC pathway
Source: Mol Carcinog. 2019 Aug 9;58(11):2026–39. doi: 10.1002/mc.23095 (PMC6851884; doi:10.1002/mc.23095)
Supplement: Supplementary file 1 — Supplementary information [file MC-58-2026-s001.doc]

Table S1. Primer sequences of the target genes

| Genes | Forward primer | Reverse primer |
| --- | --- | --- |
| CPA4 | 5’-AGGTGGATACTGTTCATTGGGG-3’ | 5’-TTGCTGATCTCGTCTCCATTTC-3’ |
| GAPDH | 5’-TGACTTCAACAGCGACACCCA-3’ | 5’-CACCCTGTTGCTGTAGCCAAA-3’ |
