# Supplementary material for: Downregulation of CPA4 inhibits non small–cell lung cancer growth by suppressing the AKT/c‐MYC pathway
Source: Mol Carcinog. 2019 Aug 9;58(11):2026–39. doi: 10.1002/mc.23095 (PMC6851884; doi:10.1002/mc.23095)
Supplement: Supplementary file 2 — Supplementary information [file MC-58-2026-s002.doc]

Table S4. Differentially expressed genes in H1299-shCPA4 cells

| **probeSet** | **Gene Symbol** | **Fold change** | **P-value** |
| --- | --- | --- | --- |
| 11721874_at | IFIT2 | 24.968733 | 3.96216E-07 |
| 11756820_a_at | IFIT1 | 18.031782 | 8.58815E-08 |
| 11731407_x_at | IFIT3 | 10.875454 | 0.000363544 |
| 11758198_s_at | DDIT3 | -9.567241 | 9.09429E-06 |
| 11757044_x_at | HILPDA | -9.014507 | 4.41065E-06 |
| 11757367_s_at | MYC | -8.769676 | 2.8182E-05 |
| 11727906_at | MMP10 | -8.474572 | 4.04585E-05 |
| 11715441_a_at | CTGF | -8.191285 | 2.71833E-06 |
| 11763492_a_at | NRN1 | -8.012061 | 9.56257E-07 |
| 11715493_a_at | CYR61 | -7.77964 | 1.39942E-07 |
| 11723050_a_at | HSPA6 | -7.627095 | 8.54871E-06 |
| 11721917_a_at | ANKRD37 | -7.5987177 | 1.22937E-05 |
| 11748003_a_at | IFI44 | 7.433438 | 5.57901E-05 |
| 11729119_a_at | HSPA1A | -6.550447 | 1.53752E-07 |
| 11729119_a_at | HSPA1B | -6.550447 | 1.53752E-07 |
| 11748793_a_at | ASNS | -6.480583 | 4.41353E-06 |
| 11716710_a_at | ADM | -6.475699 | 1.19829E-05 |
| 11746283_s_at | LOC730755 | -6.330271 | 0.00016816 |
| 11746283_s_at | RRM2 | -6.330271 | 0.00016816 |
| 11715670_a_at | IFITM1 | 6.286618 | 0.000160693 |
| 11734659_a_at | FOS | -5.9487243 | 0.000142427 |
| 11721090_at | THBS1 | -5.7537556 | 4.96025E-06 |
| 11718141_at | FOSL1 | -5.7484703 | 0.0005809 |
| 11753421_a_at | CITED2 | -5.7418118 | 5.82515E-06 |
| 11763017_at | HSPD1 | -5.6453786 | 1.15268E-06 |
| 11746506_a_at | SPP1 | -5.4480886 | 3.35098E-06 |
| 11757166_at | SNORD14D | -5.2829747 | 1.08679E-05 |
| 11757166_at | SNORD14C | -5.2829747 | 1.08679E-05 |
| 11757166_at | HSPA8 | -5.2829747 | 1.08679E-05 |
| 11756330_a_at | DDX58 | 5.2014594 | 8.06916E-06 |
| 11716630_a_at | ANGPTL4 | -5.0034037 | 1.37151E-06 |
| 11719292_a_at | ITGB1BP1 | 5.0019007 | 0.000903998 |
| 11744236_a_at | DDX60 | 4.976981 | 4.61811E-05 |
| 11715445_a_at | DNAJB1 | -4.963529 | 8.52319E-09 |
| 11721876_s_at | SLC7A11 | -4.923607 | 9.15662E-06 |
| 11756553_a_at | GUK1 | -4.854326 | 5.53498E-07 |
| 11745021_a_at | HSPA7 | -4.8288803 | 2.16778E-07 |
| 11759123_at | TMEM150C | 4.8164105 | 0.00216785 |
| 11759123_at | RPS6P6 | 4.8164105 | 0.00216785 |
| 11721435_at | STC2 | -4.765725 | 4.89731E-05 |
| 11739258_a_at | VWA5A | 4.7649198 | 6.73472E-05 |
| 11759728_at | SEC24A | -4.7337728 | 0.000204538 |
| 11722462_a_at | TNKS2 | 4.701542 | 2.16337E-05 |
| 11746248_x_at | MANSC1 | 4.633443 | 5.40534E-06 |
| 11728076_at | HDAC9 | -4.4917207 | 0.000124407 |
| 11751342_a_at | NDRG1 | -4.4165664 | 3.5015E-06 |
| 11753988_a_at | SPRY2 | -4.3957806 | 1.9374E-05 |
| 11715514_a_at | HERPUD1 | -4.3868904 | 3.54849E-06 |
| 11719024_at | ZNF652 | 4.3485126 | 2.54961E-05 |
| 11719849_s_at | UBE2S | -4.334678 | 7.99571E-06 |
| 11726364_x_at | OASL | 4.3308573 | 0.000104346 |
| 11743972_a_at | DDIT4 | -4.3006425 | 6.2286E-06 |
| 11754360_a_at | KRTAP2-4 | -4.253848 | 6.84284E-07 |
| 11738414_a_at | VEGFA | -4.228056 | 0.000723785 |
| 11733695_a_at | UBE2C | -4.2190623 | 1.89113E-07 |
| 11726677_a_at | PSMB9 | 4.18992 | 3.56342E-05 |
| 11717374_at | DNAJC3 | -4.184636 | 0.000259316 |
| 11732585_a_at | IL1RAP | -4.0782275 | 9.49011E-05 |
| 11722231_at | PDP1 | -4.031318 | 3.76191E-05 |
| 11759131_at | HIST1H2AG | 3.9952855 | 6.11886E-05 |
| 11759131_at | HIST1H2AI | 3.9952855 | 6.11886E-05 |
| 11759131_at | HIST1H2AH | 3.9952855 | 6.11886E-05 |
| 11759131_at | HIST1H2AM | 3.9952855 | 6.11886E-05 |
| 11759131_at | HIST1H2AL | 3.9952855 | 6.11886E-05 |
| 11756098_a_at | SYTL5 | -3.9641168 | 0.000220326 |
| 11731768_x_at | ARSB | 3.9086761 | 2.31265E-05 |
| 11723419_a_at | COL9A3 | 3.9032075 | 3.58626E-06 |
| 11752940_a_at | EGR1 | -3.8848062 | 1.12027E-05 |
| 11724021_at | HIST1H2BD | 3.8782067 | 3.37414E-05 |
| 11717475_a_at | CHGB | 3.8487153 | 2.13885E-06 |
| 11728563_at | FOXD1 | -3.8225315 | 1.03876E-05 |
| 11721307_at | PHLDA1 | -3.8033655 | 2.12608E-07 |
| 11737749_a_at | SGK1 | -3.8011343 | 1.3557E-05 |
| 11744434_a_at | PARP9 | 3.7823877 | 0.000268286 |
| 11725765_a_at | COL11A1 | 3.7683115 | 8.08204E-05 |
| 11758781_at | EMP1 | -3.7669022 | 4.89187E-06 |
| 11741223_s_at | FAM198B | -3.7033005 | 0.000192436 |
| 11715659_s_at | ERRFI1 | -3.6935298 | 1.49796E-06 |
| 11715431_a_at | TMBIM1 | -3.6909318 | 6.38278E-06 |
| 11754443_a_at | GTF2F1 | -3.6839488 | 5.9882E-06 |
| 11747458_a_at | MOK | -3.6692302 | 5.47115E-06 |
| 11750123_a_at | CKAP2L | -3.6678405 | 2.85799E-05 |
| 11749311_a_at | GRPEL1 | -3.6207395 | 1.06335E-05 |
| 11739303_a_at | DLC1 | -3.617273 | 7.88389E-05 |
| 11755777_a_at | RECK | 3.6066263 | 0.000390801 |
| 11756138_a_at | HK2 | -3.6063893 | 2.78826E-06 |
| 11741315_a_at | PLSCR4 | 3.6061082 | 0.000107922 |
| 11723668_at | SEMA3C | -3.604419 | 1.46193E-05 |
| 11732297_at | KIAA1715 | -3.6012151 | 3.32425E-05 |
| 11718576_s_at | NET1 | -3.5969505 | 0.000511952 |
| 11719960_a_at | CTBP1 | -3.5967371 | 2.5991E-05 |
| 11718037_x_at | MDK | 3.5952518 | 0.000232942 |
| 11725931_at | HSPA5 | -3.5714748 | 9.70548E-05 |
| 11720833_at | YEATS4 | -3.5551002 | 0.00023414 |
| 11726356_at | ENPP1 | 3.546553 | 0.000200057 |
| 11724848_a_at | DIXDC1 | 3.5444772 | 7.01711E-05 |
| 11728054_a_at | DIAPH3 | -3.543599 | 0.000223988 |
| 11715370_s_at | LGALS3BP | 3.5412521 | 2.99226E-05 |
| 11753130_at | TM4SF1 | -3.5364845 | 1.05771E-07 |
| 11727145_s_at | KLF11 | -3.5075338 | 0.000425035 |
| 11745608_a_at | WDR1 | -3.5027716 | 2.63404E-05 |
| 11735544_x_at | C8orf44 | 3.490058 | 8.67413E-06 |
| 11723747_a_at | FAM64A | -3.485074 | 0.000719521 |
| 11727102_a_at | IGF2BP1 | -3.4845428 | 0.001012967 |
| 11718394_at | JUN | -3.4640534 | 5.35967E-05 |
| 11719475_a_at | BEX1 | 3.4615514 | 0.000211818 |
| 11736669_s_at | SPRY4 | -3.4612436 | 0.000506791 |
| 11729450_at | PCLO | 3.4581923 | 1.16836E-05 |
| 11744667_a_at | DENND5B | -3.4248953 | 6.74051E-05 |
| 11725403_at | TM4SF18 | -3.419471 | 1.17092E-05 |
| 11743010_at | NFIL3 | -3.407143 | 1.1154E-06 |
| 11748314_a_at | CDC20 | -3.36648 | 8.28372E-06 |
| 11723290_a_at | CRISPLD1 | 3.3570318 | 2.42088E-05 |
| 11734982_a_at | GPRASP1 | 3.3509536 | 0.000740435 |
| 11744068_x_at | APOE | 3.3311865 | 1.04128E-06 |
| 11730049_at | CA13 | 3.3300233 | 8.50992E-05 |
| 11730049_at | LOC100507258 | 3.3300233 | 8.50992E-05 |
| 11748002_a_at | HPCAL1 | -3.3121514 | 7.06554E-06 |
| 11733491_a_at | SLC44A5 | 3.3015208 | 2.31092E-05 |
| 11733809_s_at | ARMCX5-GPRASP2 | 3.2906125 | 0.000787481 |
| 11733809_s_at | GPRASP2 | 3.2906125 | 0.000787481 |
| 11719792_at | GPRC5A | -3.2786505 | 3.44104E-05 |
| 11718106_s_at | IPO5 | -3.2725904 | 1.19944E-05 |
| 11716094_a_at | KLF6 | -3.264447 | 6.05491E-07 |
| 11727286_a_at | ZNF323 | 3.2597358 | 6.77237E-05 |
| 11723907_a_at | LARP1B | -3.2590423 | 0.000139103 |
| 11716895_s_at | ISG15 | 3.255726 | 6.90139E-05 |
| 11754434_a_at | CCND3 | 3.2519715 | 2.32015E-06 |
| 11725496_a_at | AGPAT9 | -3.2503572 | 0.000140657 |
| 11735148_a_at | SOCS2 | -3.247393 | 0.000898021 |
| 11724477_a_at | DKK1 | -3.2364383 | 8.78933E-05 |
| 11755374_a_at | HERC5 | 3.2258842 | 0.000378026 |
| 11758211_s_at | PM20D2 | -3.225059 | 6.93656E-05 |
| 11718936_s_at | MMD | -3.2223537 | 4.74065E-05 |
| 11728776_s_at | VDR | 3.2139556 | 0.001820364 |
| 11754629_s_at | SLC16A6 | 3.186118 | 0.000296136 |
| 11720067_a_at | APH1B | 3.1845717 | 0.00072402 |
| 11722826_a_at | NCAPG | -3.1814578 | 9.96251E-06 |
| 11722406_at | PFKFB4 | -3.1727378 | 7.17608E-08 |
| 11733816_a_at | RPL22L1 | -3.1516142 | 2.83741E-06 |
| 11742945_a_at | EBNA1BP2 | -3.1493812 | 1.16289E-05 |
| 11752177_a_at | SIAH1 | -3.1393242 | 5.63259E-05 |
| 11762010_a_at | GRAMD1B | -3.1392314 | 0.00058886 |
| 11756547_a_at | CLU | 3.1371636 | 5.11131E-05 |
| 11719344_a_at | ATF3 | -3.1317987 | 0.00053367 |
| 11737440_a_at | BMF | 3.131657 | 4.10013E-06 |
| 11753976_a_at | ZMYM3 | 3.1274939 | 5.14976E-05 |
| 11757725_a_at | NHP2 | -3.1241398 | 4.3847E-05 |
| 11757522_s_at | C6orf47 | 3.119753 | 3.10716E-05 |
| 11722436_a_at | GOSR2 | -3.1181095 | 9.67246E-05 |
| 11723854_at | SAMD9 | 3.1129913 | 0.000517226 |
| 11725045_a_at | UAP1 | -3.1062455 | 9.89566E-06 |
| 11739072_at | EPDR1 | 3.1006768 | 2.48231E-05 |
| 11724786_s_at | C14orf129 | 3.100351 | 3.30586E-05 |
| 11736244_s_at | HIST1H2AC | 3.0969923 | 7.98774E-06 |
| 11732318_a_at | KHDRBS3 | -3.0938058 | 1.79002E-05 |
| 11758788_at | PHF5A | -3.0902302 | 0.00050144 |
| 11720527_at | LTBP2 | 3.089039 | 1.39009E-05 |
| 11733981_a_at | PDK1 | -3.0834575 | 0.000431903 |
| 11725277_a_at | TRIM6 | 3.0709302 | 0.000356936 |
| 11723863_a_at | DOCK10 | 3.0696135 | 9.29185E-05 |
| 11718076_at | MAPKAPK3 | -3.0684764 | 1.94003E-06 |
| 11717665_a_at | TSPYL4 | 3.064076 | 0.000154092 |
| 11722867_at | CDCP1 | -3.059113 | 9.34105E-06 |
| 11757262_at | SCARNA20 | 3.0531135 | 0.002057812 |
| 11732103_s_at | HIST1H3J | 3.050372 | 0.000887262 |
| 11732103_s_at | HIST1H3A | 3.050372 | 0.000887262 |
| 11732103_s_at | HIST1H3B | 3.050372 | 0.000887262 |
| 11732103_s_at | HIST1H3C | 3.050372 | 0.000887262 |
| 11732103_s_at | HIST1H3D | 3.050372 | 0.000887262 |
| 11732103_s_at | HIST1H3E | 3.050372 | 0.000887262 |
| 11732103_s_at | HIST1H3F | 3.050372 | 0.000887262 |
| 11732103_s_at | HIST1H3G | 3.050372 | 0.000887262 |
| 11732103_s_at | HIST1H3I | 3.050372 | 0.000887262 |
| 11750799_x_at | IL31RA | -3.0468931 | 4.85978E-06 |
| 11744793_x_at | DLGAP5 | -3.0433328 | 0.000230221 |
| 11739061_at | SLC7A5 | -3.0388532 | 0.000807835 |
| 11744718_a_at | CTHRC1 | 3.038686 | 2.47209E-05 |
| 11733923_a_at | CROT | 3.0339122 | 0.000339284 |
| 11720795_s_at | NUPL1 | -3.0324826 | 0.000290124 |
| 11748362_s_at | ABCC3 | 3.0299535 | 0.00023873 |
| 11748094_a_at | FAM3C | -3.024583 | 1.80824E-07 |
| 11736169_a_at | NDEL1 | -3.0187588 | 7.40179E-05 |
| 11716665_s_at | GDF15 | -3.0183048 | 0.000235732 |
| 11717039_a_at | GADD45A | -3.010754 | 6.48726E-05 |
| 11741745_a_at | ABL2 | -3.009765 | 0.00031769 |
| 11724711_a_at | DMXL1 | -2.9929097 | 0.000734739 |
| 11718591_at | SDF2L1 | -2.9901698 | 7.04013E-05 |
| 11747076_a_at | DBNL | -2.9772513 | 0.000324507 |
| 11731947_at | PAQR9 | 2.9512167 | 3.69195E-05 |
| 11733124_a_at | PEX19 | 2.9489067 | 3.29101E-05 |
| 11716338_a_at | INSIG1 | -2.939092 | 0.0001747 |
| 11717244_at | LSM4 | -2.9292946 | 6.25511E-06 |
| 11733348_s_at | CNOT7 | -2.9133334 | 0.000425427 |
| 11716019_at | RAB31 | -2.9119768 | 1.34834E-07 |
| 11740486_a_at | RUNX3 | -2.9104657 | 0.000520455 |
| 11739489_a_at | ATP10D | 2.9035254 | 0.000586681 |
| 11756222_a_at | NR1H4 | 2.9011133 | 2.24867E-05 |
| 11719738_at | DCP2 | 2.8985841 | 0.000383093 |
| 11757179_x_at | SNORD18C | 2.8970442 | 6.93164E-05 |
| 11757179_x_at | SNORD18A | 2.8970442 | 6.93164E-05 |
| 11757179_x_at | SNORD18B | 2.8970442 | 6.93164E-05 |
| 11757179_x_at | SNORD16 | 2.8970442 | 6.93164E-05 |
| 11757179_x_at | RPL4 | 2.8970442 | 6.93164E-05 |
| 11718036_at | PPIL1 | -2.886753 | 6.07041E-05 |
| 11744708_a_at | LYRM1 | -2.8823218 | 2.53031E-05 |
| 11727226_at | NAALAD2 | 2.871192 | 0.001139838 |
| 11739305_a_at | WRB | 2.8684692 | 4.95389E-05 |
| 11759429_a_at | KLF7 | -2.8578827 | 1.06828E-05 |
| 11721398_a_at | DOT1L | -2.8479328 | 4.0585E-05 |
| 11752572_a_at | SLC2A1 | -2.8365462 | 6.45498E-06 |
| 11725746_a_at | PITPNC1 | -2.8157003 | 1.27297E-05 |
| 11721113_x_at | RPL41 | 2.8076534 | 6.72679E-07 |
| 11754524_x_at | TFPI2 | -2.8045456 | 8.30627E-05 |
| 11715957_s_at | PEPD | -2.798967 | 0.000130789 |
| 11731358_at | ADAMTS3 | 2.7978067 | 0.000334457 |
| 11727666_at | TWIST1 | -2.794304 | 2.51723E-06 |
| 11727642_a_at | TRERF1 | 2.7934077 | 0.00152566 |
| 11753434_a_at | SLC3A2 | -2.7905266 | 7.7895E-06 |
| 11720571_a_at | PHF15 | -2.7831743 | 7.46074E-05 |
| 11726611_x_at | MAFF | -2.7824845 | 3.39959E-05 |
| 11752142_a_at | FLNA | -2.7668593 | 0.00127919 |
| 11719474_a_at | IMP4 | -2.757029 | 3.45088E-05 |
| 11728991_a_at | IGDCC4 | 2.7440014 | 0.003982156 |
| 11758509_s_at | ENPEP | 2.7419674 | 0.000321259 |
| 11729169_a_at | DUSP10 | -2.7335572 | 0.001979784 |
| 11717367_at | ZC3HAV1 | 2.7330787 | 2.02604E-05 |
| 11724173_at | SDSL | 2.7299125 | 7.93503E-05 |
| 11722442_a_at | NUF2 | -2.7264137 | 2.19218E-06 |
| 11720721_s_at | RRAS2 | -2.7176502 | 9.72789E-05 |
| 11716637_at | EMC7 | -2.7141378 | 0.000272942 |
| 11759656_a_at | KLHL7 | -2.7136774 | 0.000155557 |
| 11717201_at | DNTTIP1 | -2.7060275 | 1.35166E-05 |
| 11720978_at | WARS2 | -2.70478 | 0.000169486 |
| 11748907_a_at | RARRES3 | 2.7032323 | 7.21652E-05 |
| 11757459_s_at | WIPF1 | -2.6965814 | 0.000142081 |
| 11727063_a_at | MIR3658 | -2.686838 | 0.000108833 |
| 11727063_a_at | UCK2 | -2.686838 | 0.000108833 |
| 11719354_s_at | SH3BP5 | -2.686303 | 4.4495E-05 |
| 11715675_a_at | CCNG1 | -2.683785 | 7.33447E-05 |
| 11755905_a_at | ST8SIA5 | -2.6827796 | 0.000519244 |
| 11721024_a_at | IL11RA | 2.6824188 | 7.46356E-05 |
| 11718723_at | ARRDC3 | -2.6814165 | 0.001711315 |
| 11729623_a_at | KIF2C | -2.6810422 | 0.000248864 |
| 11744549_s_at | COX20 | -2.6660364 | 3.64483E-06 |
| 11757586_a_at | SLC27A5 | -2.6660225 | 6.02942E-06 |
| 11733530_at | C1RL | 2.6573524 | 0.000692601 |
| 11721994_s_at | UBE2L6 | 2.6556017 | 1.55277E-05 |
| 11755043_a_at | PION | 2.650568 | 0.000247859 |
| 11725347_a_at | TFDP1 | -2.6483505 | 0.00070278 |
| 11719811_a_at | TRIB3 | -2.6451519 | 4.41739E-06 |
| 11719608_a_at | BCAR3 | -2.6446605 | 0.000381661 |
| 11716168_s_at | MRFAP1L1 | 2.6394253 | 4.34663E-06 |
| 11718518_at | KCMF1 | -2.6332014 | 7.5557E-06 |
| 11728189_a_at | CXCR4 | -2.632856 | 0.000100399 |
| 11733936_a_at | PAIP1 | 2.6298928 | 0.000504633 |
| 11757192_a_at | C17orf76-AS1 | -2.6297052 | 0.000196511 |
| 11719371_a_at | GYS1 | -2.6281133 | 0.000395882 |
| 11727815_at | UBASH3B | -2.6174798 | 0.000218697 |
| 11744091_s_at | ATXN7L3B | 2.616533 | 0.001530977 |
| 11741042_a_at | PSMA1 | 2.6142068 | 5.95866E-05 |
| 11745681_a_at | FUT11 | -2.610031 | 7.26832E-05 |
| 11728907_at | B3GALTL | 2.6058688 | 0.001857411 |
| 11737847_a_at | MR1 | 2.602406 | 0.000171191 |
| 11722380_at | RPS10-NUDT3 | -2.6016667 | 0.000102903 |
| 11722380_at | NUDT3 | -2.6016667 | 0.000102903 |
| 11715795_at | MANF | -2.6007903 | 1.32084E-06 |
| 11753427_a_at | RND3 | -2.599286 | 5.2204E-08 |
| 11733642_s_at | NECAP2 | 2.5976245 | 1.46859E-05 |
| 11762443_at | HIPK2 | -2.5968997 | 2.35635E-05 |
| 11716573_a_at | KDM3A | -2.5946562 | 8.3129E-05 |
| 11755748_s_at | PLAC8 | -2.5863044 | 3.95751E-05 |
| 11728302_at | TGFA | -2.586097 | 7.30255E-05 |
| 11720203_a_at | AURKB | -2.5847769 | 4.69001E-05 |
| 11716435_a_at | RAPGEF1 | -2.5836139 | 2.32837E-05 |
| 11760217_a_at | PIK3CB | -2.5826342 | 0.000445541 |
| 11763188_a_at | SNX5 | -2.581881 | 6.62764E-06 |
| 11754548_a_at | FAM82B | -2.5814219 | 0.000341954 |
| 11744219_at | G0S2 | -2.5789285 | 1.27116E-06 |
| 11720637_x_at | CXADR | 2.5763814 | 0.000663686 |
| 11730888_a_at | USF2 | -2.5707006 | 2.13573E-05 |
| 11740496_x_at | ST3GAL2 | 2.5690203 | 3.72188E-05 |
| 11752946_a_at | MAPRE2 | 2.5679836 | 2.62975E-05 |
| 11742890_at | MND1 | -2.5653656 | 9.61163E-05 |
| 11747334_a_at | DDX39A | -2.5646112 | 0.000282411 |
| 11724346_a_at | IFIH1 | 2.5626297 | 0.000445812 |
| 11745739_a_at | SCOC | -2.5607903 | 2.27841E-05 |
| 11720881_a_at | EHD4 | -2.5588334 | 4.34118E-05 |
| 11721143_a_at | MKI67 | -2.558107 | 0.000249949 |
| 11762344_at | RPS18 | -2.5552886 | 0.006449428 |
| 11716139_s_at | SMS | -2.5541666 | 4.68338E-05 |
| 11759258_at | HIST2H2AC | -2.552534 | 2.3526E-06 |
| 11733374_s_at | ZNF23 | -2.548513 | 0.001234868 |
| 11756293_a_at | UFSP2 | -2.547443 | 0.000192025 |
| 11747500_a_at | AP1M1 | -2.5439444 | 0.000364491 |
| 11744737_a_at | RTKN2 | -2.5427368 | 0.00144238 |
| 11760244_a_at | CARD8 | -2.5412583 | 0.000244195 |
| 11724612_a_at | DYRK2 | -2.5405505 | 3.56567E-05 |
| 11744054_a_at | ZNFX1 | 2.5379465 | 5.58136E-05 |
| 11757197_s_at | HNRNPU-AS1 | 2.537789 | 0.000524268 |
| 11728507_at | LY96 | 2.537163 | 8.3E-05 |
| 11756742_a_at | AGPAT5 | -2.5363379 | 0.00027109 |
| 11726289_at | GRAMD3 | 2.5331645 | 0.000268631 |
| 11722909_a_at | GATS | 2.5326905 | 1.32528E-05 |
| 11759069_at | CXXC4 | 2.5319343 | 0.000471401 |
| 11723010_a_at | KIF20A | -2.5274248 | 3.85213E-05 |
| 11734691_a_at | TAF11 | 2.5258715 | 3.9455E-05 |
| 11717481_s_at | FKBP10 | -2.522165 | 0.002933186 |
| 11718201_at | HEY1 | -2.5179484 | 0.001708762 |
| 11720064_a_at | LOC100652973 | 2.5164545 | 1.53576E-05 |
| 11720064_a_at | GLIPR2 | 2.5164545 | 1.53576E-05 |
| 11759644_at | OSMR | -2.515905 | 7.35884E-05 |
| 11715563_s_at | SCD | -2.515883 | 0.000612229 |
| 11715722_a_at | GOLM1 | 2.515181 | 2.69598E-05 |
| 11750722_a_at | GPHN | -2.5148613 | 0.000192838 |
| 11727331_at | SPAG4 | -2.5112069 | 3.23552E-06 |
| 11743084_s_at | BNIP3 | -2.50697 | 1.76475E-05 |
| 11718901_at | TGFBR3 | 2.5065274 | 0.000125477 |
| 11717448_a_at | MRPS2 | -2.5063527 | 1.72376E-06 |
| 11757903_s_at | COL4A5 | 2.5037487 | 0.000415622 |
| 11720247_a_at | CEP55 | -2.5022945 | 0.000396167 |
| 11721851_x_at | CHCHD6 | -2.4994562 | 6.88019E-05 |
| 11746373_s_at | RRN3P2 | -2.497536 | 0.001926437 |
| 11746373_s_at | RRN3P1 | -2.497536 | 0.001926437 |
| 11748534_a_at | DDAH1 | -2.4961362 | 0.000288779 |
| 11722791_s_at | MATN2 | 2.4949985 | 0.000662303 |
| 11722791_s_at | LOC100506558 | 2.4949985 | 0.000662303 |
| 11753586_s_at | CXADRP2 | 2.494586 | 0.00116699 |
| 11753586_s_at | CXADRP3 | 2.494586 | 0.00116699 |
| 11726320_at | ERO1L | -2.4923522 | 5.5302E-05 |
| 11715665_a_at | PSMB8 | 2.492303 | 0.000427449 |
| 11716939_a_at | HMOX1 | -2.4875207 | 0.000108761 |
| 11717561_s_at | DTX3L | 2.482586 | 0.000765627 |
| 11730774_at | TMEM14A | 2.4822068 | 7.37681E-06 |
| 11716179_at | C5orf15 | 2.481275 | 7.01984E-05 |
| 11716151_a_at | PMEPA1 | -2.4787302 | 2.78998E-05 |
| 11748516_a_at | NAP1L5 | 2.4786065 | 0.000168804 |
| 11726498_at | LIF | -2.477478 | 9.28702E-05 |
| 11743123_a_at | TLN1 | 2.4752934 | 0.000152693 |
| 11722530_a_at | CALD1 | 2.4749932 | 0.000170998 |
| 11720503_a_at | ALDH3A2 | 2.4735012 | 2.83045E-05 |
| 11758403_s_at | RAPH1 | -2.4730065 | 0.000578605 |
| 11727916_a_at | C2orf18 | -2.4706545 | 0.000141391 |
| 11728984_a_at | MTHFD1L | -2.4702463 | 9.48273E-06 |
| 11725448_at | PLD6 | -2.4688332 | 0.000156037 |
| 11723578_a_at | C6orf228 | -2.466858 | 0.002512327 |
| 11723250_a_at | EML2 | -2.465516 | 0.001130999 |
| 11721654_at | AGPAT4 | 2.4651067 | 0.000177102 |
| 11727510_at | UBN2 | 2.4645152 | 2.03878E-05 |
| 11741504_a_at | NREP | 2.4642017 | 0.000472552 |
| 11716851_at | IMPAD1 | -2.4626627 | 0.000135456 |
| 11735707_x_at | NABP2 | -2.457242 | 0.000187271 |
| 11722069_a_at | SKA1 | -2.4568417 | 5.05385E-05 |
| 11733938_x_at | LYPLA2 | 2.4555788 | 0.000597612 |
| 11734793_at | SNAPC1 | -2.4542017 | 9.66767E-06 |
| 11720277_a_at | OLFML2A | 2.4532335 | 0.000405233 |
| 11724454_at | SLC22A4 | 2.453077 | 0.000352008 |
| 11723367_a_at | RAB26 | 2.452895 | 4.81316E-05 |
| 11746946_a_at | AGGF1 | 2.4525213 | 0.000345263 |
| 11720541_at | LGALSL | -2.4521635 | 0.000311579 |
| 11758030_s_at | FOXG1 | -2.4517608 | 3.03368E-05 |
| 11754352_a_at | RNF213 | 2.4474418 | 6.76108E-05 |
| 11732331_s_at | ARL4C | -2.4454715 | 3.0414E-05 |
| 11719598_s_at | CDCA3 | -2.4450169 | 0.000422674 |
| 11758867_at | SFT2D3 | -2.444935 | 0.000744295 |
| 11758867_at | WDR33 | -2.444935 | 0.000744295 |
| 11758854_at | ATL3 | 2.4445148 | 0.000437093 |
| 11721702_a_at | CMTM7 | -2.4444938 | 6.61923E-05 |
| 11720508_at | FBN1 | 2.4444537 | 0.002147329 |
| 11759623_at | C11orf71 | 2.4433117 | 0.000806748 |
| 11756773_a_at | FRMD5 | -2.4425278 | 0.001875542 |
| 11721185_at | MEGF8 | 2.4371443 | 1.41141E-05 |
| 11728759_a_at | PPHLN1 | 2.4370747 | 0.00045507 |
| 11716086_a_at | PXDN | 2.4368782 | 0.00018277 |
| 11731059_a_at | NUP35 | -2.435951 | 0.000946048 |
| 11722572_at | CCNA2 | -2.4358597 | 1.87736E-05 |
| 11744347_a_at | MYO10 | -2.429708 | 6.21928E-05 |
| 11735385_s_at | DACT1 | -2.4287505 | 9.16542E-05 |
| 11745843_a_at | CDK1 | -2.4282975 | 3.33831E-06 |
| 11717129_s_at | SAR1A | 2.4275067 | 8.40605E-06 |
| 11748909_a_at | BNIP3L | -2.4265764 | 0.000530446 |
| 11721473_a_at | HCCS | 2.4246807 | 0.000676892 |
| 11716554_a_at | HLA-DMA | 2.420959 | 0.001191842 |
| 11730102_at | SCN9A | 2.420003 | 0.000290083 |
| 11755606_x_at | IFI30 | 2.418135 | 0.000206148 |
| 11716793_a_at | CCNB2 | -2.4167874 | 0.000706801 |
| 11718842_a_at | C16orf62 | -2.4148552 | 3.4489E-05 |
| 11751206_s_at | RAB3GAP2 | -2.4143631 | 0.000149852 |
| 11751206_s_at | AURKAPS1 | -2.4143631 | 0.000149852 |
| 11723033_at | NOV | 2.4107833 | 0.000787204 |
| 11724360_s_at | BTRC | 2.4101546 | 2.75999E-05 |
| 11721632_a_at | TMEM97 | -2.4069483 | 0.000275765 |
| 11736685_a_at | FGF5 | -2.40627 | 1.51074E-05 |
| 11716633_a_at | TMEM50A | 2.4025133 | 0.000526963 |
| 11763767_at | MSMO1 | -2.402295 | 0.000385835 |
| 11724312_a_at | SH3BP2 | 2.4013007 | 0.000213844 |
| 11720535_a_at | MMAB | -2.3998663 | 0.000104621 |
| 11748196_a_at | ACADSB | -2.398893 | 1.36558E-05 |
| 11755290_s_at | FBXL18 | 2.3928552 | 0.000247736 |
| 11743923_at | TCFL5 | 2.3915687 | 9.05338E-05 |
| 11743923_at | DPH3P1 | 2.3915687 | 9.05338E-05 |
| 11716822_a_at | SLC9B2 | 2.3888633 | 0.000100287 |
| 11736068_x_at | NDRG4 | 2.388218 | 0.000110966 |
| 11724337_s_at | NRCAM | 2.3879151 | 0.000220356 |
| 11741897_a_at | MMP1 | -2.3878233 | 0.000114615 |
| 11734117_a_at | BTN3A3 | 2.387205 | 2.06456E-05 |
| 11723884_at | SOWAHC | -2.3862116 | 0.000102206 |
| 11728203_a_at | PHGDH | -2.3846636 | 1.86045E-05 |
| 11720369_at | RANBP6 | 2.3811684 | 0.000585648 |
| 11741015_a_at | CDCA8 | -2.380258 | 0.000172536 |
| 11751215_a_at | SLAIN1 | -2.37686 | 6.57564E-05 |
| 11749065_a_at | MCU | -2.3765414 | 7.26959E-05 |
| 11720731_a_at | SUMF1 | 2.3749468 | 0.000128387 |
| 11719141_a_at | INSIG2 | -2.3739088 | 4.96073E-06 |
| 11760320_at | RPL31 | 2.3724601 | 0.000405371 |
| 11747201_a_at | IDS | -2.3688629 | 0.000732821 |
| 11722715_at | STK35 | -2.3688147 | 0.000178871 |
| 11742735_a_at | NUSAP1 | -2.3680837 | 0.000102279 |
| 11731466_a_at | CTSC | -2.367021 | 2.41896E-06 |
| 11725209_at | LETM1 | -2.3665886 | 6.68531E-05 |
| 11736931_s_at | FAM72A | -2.3661022 | 9.96317E-06 |
| 11736931_s_at | FAM72B | -2.3661022 | 9.96317E-06 |
| 11736931_s_at | FAM72D | -2.3661022 | 9.96317E-06 |
| 11717863_a_at | DUSP5 | -2.3657393 | 1.62106E-05 |
| 11722253_a_at | NEK2 | -2.3654587 | 0.000748626 |
| 11717986_a_at | APOC1 | 2.3610635 | 9.99288E-05 |
| 11749671_a_at | NPAS2 | -2.360662 | 0.000190243 |
| 11727339_a_at | ZBTB2 | -2.3597763 | 0.000183722 |
| 11720209_at | IRF9 | 2.3555057 | 6.21956E-05 |
| 11749287_a_at | ARL2BP | 2.354432 | 8.29731E-06 |
| 11715503_a_at | LASP1 | -2.3540225 | 0.000126311 |
| 11752387_a_at | ETS1 | -2.35261 | 0.000709474 |
| 11739610_a_at | CALML4 | -2.3517659 | 0.000327069 |
| 11738335_x_at | TP53 | 2.3497064 | 0.000758896 |
| 11720129_a_at | COMTD1 | -2.3491716 | 0.002713906 |
| 11730856_s_at | HMGN3 | 2.3456194 | 5.4939E-05 |
| 11717041_at | MTMR4 | -2.3455386 | 1.0292E-05 |
| 11718058_a_at | TYMS | -2.343871 | 8.56261E-06 |
| 11720662_at | TSR3 | -2.3400068 | 0.000397482 |
| 11719099_at | ISOC1 | -2.3394504 | 2.29148E-06 |
| 11748155_a_at | ZNF670 | -2.3392687 | 0.000905649 |
| 11748155_a_at | ZNF670-ZNF695 | -2.3392687 | 0.000905649 |
| 11748155_a_at | ZNF695 | -2.3392687 | 0.000905649 |
| 11718751_at | NPR2 | 2.3386474 | 0.000298715 |
| 11749208_a_at | ELL2 | -2.3369884 | 0.000906751 |
| 11726276_at | SNN | 2.3328178 | 0.000156831 |
| 11730533_a_at | COL13A1 | -2.3321 | 3.1006E-06 |
| 11755735_s_at | GTPBP6 | -2.331016 | 0.00036785 |
| 11727023_at | TMEM64 | -2.3305254 | 0.00015828 |
| 11739809_at | TMEM156 | -2.3297215 | 0.000930514 |
| 11758773_at | PTPLB | -2.3294125 | 0.000107867 |
| 11718325_at | SESN2 | -2.3292098 | 0.001547004 |
| 11725155_at | FJX1 | -2.3289018 | 0.000476453 |
| 11746094_a_at | MPP4 | -2.327146 | 0.000438995 |
| 11715530_a_at | CCT6A | -2.3263848 | 3.61184E-05 |
| 11739503_at | ABCA1 | 2.3251119 | 2.2438E-05 |
| 11754428_a_at | ZFYVE26 | 2.3226368 | 0.000227311 |
| 11724573_at | APPBP2 | 2.3225389 | 0.001006351 |
| 11751791_a_at | CCDC80 | 2.3208346 | 0.000429937 |
| 11718469_s_at | USP33 | 2.3195834 | 0.000218019 |
| 11752448_a_at | P4HB | -2.3185766 | 6.91606E-05 |
| 11717196_a_at | MEST | 2.316275 | 0.000132965 |
| 11736309_a_at | CSNK1A1 | -2.3144212 | 0.001118999 |
| 11741105_a_at | FHL2 | -2.3143961 | 0.000176574 |
| 11716329_s_at | GJA1 | 2.3136506 | 0.00049503 |
| 11717043_at | C20orf11 | -2.3100114 | 0.000227732 |
| 11725574_a_at | NEDD4 | 2.3093603 | 0.000105397 |
| 11717927_at | FOXK2 | -2.3081555 | 6.01775E-06 |
| 11716294_a_at | CARS | -2.3075118 | 7.03038E-06 |
| 11755190_a_at | POLR1D | -2.3043709 | 8.05159E-05 |
| 11760576_a_at | PXK | -2.3001723 | 0.000633778 |
| 11727287_at | C5orf34 | 2.295029 | 6.85041E-05 |
| 11743917_a_at | FKBP5 | 2.294267 | 0.000253281 |
| 11719130_s_at | POLR3D | -2.2936916 | 0.000122027 |
| 11741413_a_at | ZNF664-FAM101A | -2.2932599 | 0.000464423 |
| 11741413_a_at | FAM101A | -2.2932599 | 0.000464423 |
| 11720320_at | AK2 | -2.293083 | 1.97811E-05 |
| 11731992_x_at | TROAP | -2.2924726 | 0.000232091 |
| 11757047_x_at | ATF4 | -2.291086 | 1.18827E-05 |
| 11716115_s_at | ZFP36L1 | -2.2879236 | 0.000615275 |
| 11724917_at | TMTC3 | 2.2865236 | 0.00974435 |
| 11754260_a_at | NBR2 | 2.284208 | 0.00232248 |
| 11719707_a_at | DHRS13 | -2.2820697 | 0.000120732 |
| 11745775_a_at | LOC100507575 | 2.2807667 | 2.03737E-06 |
| 11745775_a_at | LIPA | 2.2807667 | 2.03737E-06 |
| 11740481_x_at | PAQR5 | -2.2792704 | 0.001842565 |
| 11721668_at | CDT1 | -2.278442 | 0.000222491 |
| 11725885_a_at | CNEP1R1 | 2.277957 | 7.4576E-06 |
| 11731897_a_at | PTHLH | -2.277199 | 0.002568673 |
| 11753880_x_at | CDKN3 | -2.2764814 | 0.000866964 |
| 11725385_at | UGCG | -2.2757277 | 0.000241405 |
| 11718356_at | ERP44 | 2.2752419 | 0.000170572 |
| 11738080_x_at | ANKRD20A4 | -2.2737243 | 0.000361388 |
| 11738080_x_at | ANKRD20A3 | -2.2737243 | 0.000361388 |
| 11738080_x_at | ANKRD20A1 | -2.2737243 | 0.000361388 |
| 11738080_x_at | ANKRD20A2 | -2.2737243 | 0.000361388 |
| 11717551_a_at | LOXL1 | -2.271314 | 8.93711E-05 |
| 11735294_a_at | LRRK2 | 2.2713103 | 0.000886521 |
| 11724358_s_at | MYO5A | 2.2711935 | 0.000998361 |
| 11727158_a_at | STRBP | -2.2702038 | 0.000431301 |
| 11727715_at | ITGA10 | -2.2694664 | 0.00010893 |
| 11725978_at | NCBP1 | -2.2682664 | 8.85278E-05 |
| 11734614_a_at | GABRE | 2.2668605 | 0.000403137 |
| 11734614_a_at | MIR224 | 2.2668605 | 0.000403137 |
| 11734614_a_at | MIR452 | 2.2668605 | 0.000403137 |
| 11729949_at | KLHL14 | 2.2654011 | 0.001399777 |
| 11730853_s_at | P4HA1 | -2.2638414 | 4.62393E-05 |
| 11728565_x_at | FAM210A | -2.2625093 | 4.80467E-05 |
| 11723919_x_at | LMAN2 | -2.2595487 | 1.16149E-05 |
| 11723821_a_at | SMURF2 | -2.2587132 | 0.000830773 |
| 11744751_a_at | SERPINB9 | 2.2579904 | 0.000712144 |
| 11716535_a_at | PPT1 | 2.2579033 | 4.52826E-06 |
| 11719605_at | RRN3 | -2.2567668 | 8.25818E-05 |
| 11763310_at | MIR21 | 2.2561953 | 0.000220074 |
| 11750162_a_at | WDR45L | -2.2522054 | 0.000114788 |
| 11744522_a_at | BOD1 | -2.251962 | 4.2837E-06 |
| 11717171_at | BCL2L2 | -2.2508442 | 7.0814E-05 |
| 11743321_at | CTSO | 2.250237 | 0.000352827 |
| 11724435_a_at | TPK1 | 2.2500331 | 0.00030624 |
| 11716188_s_at | SEC13 | -2.2466853 | 0.000355988 |
| 11751651_s_at | PLSCR1 | 2.2425475 | 1.8425E-06 |
| 11751638_a_at | NT5C | -2.2410724 | 2.84243E-05 |
| 11720135_a_at | PROS1 | 2.2399452 | 2.98396E-05 |
| 11755700_a_at | TIPARP | -2.232455 | 0.000573974 |
| 11755213_a_at | KIAA1522 | 2.2308764 | 0.002944164 |
| 11744764_s_at | WHSC1 | -2.229559 | 0.000518024 |
| 11760038_a_at | GLS | -2.2286413 | 0.00055589 |
| 11731151_a_at | LIX1L | -2.2278962 | 0.000573026 |
| 11735400_a_at | BNC1 | -2.2259715 | 8.83664E-05 |
| 11718376_s_at | LOC401127 | -2.225393 | 0.000788856 |
| 11718376_s_at | WDR5 | -2.225393 | 0.000788856 |
| 11752331_s_at | SLX1A-SULT1A3 | 2.224696 | 3.42241E-06 |
| 11752331_s_at | SULT1A3 | 2.224696 | 3.42241E-06 |
| 11752331_s_at | SULT1A4 | 2.224696 | 3.42241E-06 |
| 11752331_s_at | SLX1B-SULT1A4 | 2.224696 | 3.42241E-06 |
| 11720972_at | TOP2A | -2.2238715 | 0.005458958 |
| 11763285_at | KLHDC10 | -2.2229319 | 0.001924388 |
| 11741135_s_at | CHN1 | -2.222587 | 0.000532698 |
| 11717056_a_at | CORO1C | -2.222012 | 0.000703577 |
| 11742287_a_at | CHAC1 | -2.2209795 | 0.000283002 |
| 11759219_at | HIST1H2AK | 2.2208257 | 0.00185561 |
| 11754031_s_at | CKS1B | -2.2172878 | 0.000615592 |
| 11730111_a_at | DEPDC1 | -2.212777 | 0.000311073 |
| 11753802_a_at | SRSF5 | 2.2083135 | 3.52713E-05 |
| 11752887_x_at | EHD1 | -2.2079308 | 0.000237047 |
| 11740489_at | MAPK9 | 2.2079146 | 0.006956821 |
| 11749745_a_at | SRP68 | -2.2053633 | 2.33804E-05 |
| 11758970_a_at | PVRL3 | -2.2038538 | 0.00022068 |
| 11756809_a_at | EFHC2 | 2.2016249 | 0.001803045 |
| 11718664_a_at | SLC35B2 | 2.200093 | 9.06889E-06 |
| 11718664_a_at | MIR4647 | 2.200093 | 9.06889E-06 |
| 11741629_a_at | POLR3H | -2.1974556 | 0.00143815 |
| 11737791_s_at | APOL2 | 2.1944587 | 0.000522014 |
| 11737791_s_at | APOL1 | 2.1944587 | 0.000522014 |
| 11721993_at | SLC6A6 | 2.189632 | 8.31605E-06 |
| 11724800_a_at | QPRT | 2.1888444 | 0.000409229 |
| 11720555_a_at | TSEN15 | -2.1871803 | 0.002266941 |
| 11719208_s_at | NPTN | 2.1871762 | 0.000272254 |
| 11727904_s_at | IL13RA1 | 2.1835346 | 0.00015199 |
| 11722844_a_at | ENAH | -2.1812155 | 0.000249963 |
| 11716198_a_at | UBE2Q2 | -2.179377 | 3.81057E-05 |
| 11733836_a_at | TRO | 2.176954 | 0.000444369 |
| 11745781_s_at | ZNF540 | 2.1766176 | 0.000744289 |
| 11726436_a_at | ANKRD16 | -2.1765945 | 0.001122439 |
| 11755400_s_at | STK39 | -2.1747928 | 0.000115089 |
| 11745571_a_at | CENPA | -2.1739857 | 0.000858954 |
| 11720266_a_at | TBCK | 2.166175 | 0.000172038 |
| 11759076_at | HIST1H3H | 2.1661458 | 0.000428174 |
| 11747479_x_at | MLST8 | -2.1657486 | 0.000654387 |
| 11737384_at | PCDH7 | 2.1639123 | 0.004529913 |
| 11758238_s_at | ABCC4 | -2.163868 | 0.000133058 |
| 11762576_at | BCL2L11 | 2.1635833 | 0.000168172 |
| 11721158_a_at | DRAM2 | -2.1634638 | 1.15888E-05 |
| 11724103_x_at | C8orf44-SGK3 | 2.1621234 | 1.42362E-05 |
| 11724103_x_at | SGK3 | 2.1621234 | 1.42362E-05 |
| 11718030_at | RAB11A | 2.160397 | 4.09196E-05 |
| 11718947_a_at | MPP5 | -2.1583674 | 0.000779718 |
| 11752664_a_at | S1PR5 | 2.1582289 | 0.000428269 |
| 11740114_a_at | CGN | 2.1579306 | 0.000164151 |
| 11724275_s_at | TMEM158 | -2.15554 | 3.0085E-05 |
| 11720558_a_at | GEM | -2.1537538 | 2.64123E-05 |
| 11716268_at | AK1 | 2.1537213 | 0.00064541 |
| 11725951_at | RIOK3 | -2.1525667 | 0.00027657 |
| 11739560_at | GDAP1 | 2.151224 | 2.46395E-05 |
| 11719351_s_at | GCC2 | 2.1497028 | 6.62407E-05 |
| 11739199_a_at | TMEM106C | -2.1488998 | 5.35018E-06 |
| 11736079_a_at | NEK9 | 2.1470404 | 0.000588056 |
| 11728857_at | CEP19 | 2.1462653 | 0.002275533 |
| 11734736_a_at | FAR1 | -2.1450522 | 0.001176105 |
| 11743373_a_at | C15orf23 | -2.1443832 | 5.49137E-05 |
| 11723998_at | SPC25 | -2.1431859 | 0.000331176 |
| 11718854_a_at | MRPS30 | -2.1428044 | 0.000121219 |
| 11743968_a_at | CYBA | 2.1423209 | 7.25164E-06 |
| 11721345_at | PLEK2 | 2.1421182 | 7.146E-07 |
| 11759450_x_at | DDI2 | -2.140627 | 0.000177594 |
| 11759450_x_at | RSC1A1 | -2.140627 | 0.000177594 |
| 11755874_a_at | BORA | -2.1377528 | 0.000132113 |
| 11763195_at | ARAF | 2.1356544 | 0.000316834 |
| 11754328_a_at | PIGG | 2.1340282 | 0.000216691 |
| 11737448_x_at | DNAJC22 | 2.133166 | 0.000611912 |
| 11743054_a_at | CHORDC1 | -2.1328309 | 0.000227689 |
| 11758359_s_at | SLC5A12 | 2.1318622 | 0.00016887 |
| 11754960_a_at | PRRT2 | 2.1314056 | 0.000653751 |
| 11733247_a_at | SPG21 | -2.1286988 | 2.54787E-05 |
| 11734843_x_at | MPHOSPH8 | 2.1286385 | 3.58698E-05 |
| 11716788_at | B3GNT1 | 2.1283677 | 8.86002E-05 |
| 11738060_s_at | HIST1H4L | 2.1283581 | 0.000768843 |
| 11738060_s_at | HIST1H4K | 2.1283581 | 0.000768843 |
| 11738060_s_at | HIST1H4A | 2.1283581 | 0.000768843 |
| 11738060_s_at | HIST1H4B | 2.1283581 | 0.000768843 |
| 11738060_s_at | HIST1H4E | 2.1283581 | 0.000768843 |
| 11738060_s_at | HIST1H4F | 2.1283581 | 0.000768843 |
| 11738060_s_at | HIST1H4C | 2.1283581 | 0.000768843 |
| 11738060_s_at | HIST1H4D | 2.1283581 | 0.000768843 |
| 11738060_s_at | HIST1H4I | 2.1283581 | 0.000768843 |
| 11738060_s_at | HIST1H4J | 2.1283581 | 0.000768843 |
| 11738060_s_at | HIST1H4H | 2.1283581 | 0.000768843 |
| 11738060_s_at | HIST4H4 | 2.1283581 | 0.000768843 |
| 11738060_s_at | HIST2H4A | 2.1283581 | 0.000768843 |
| 11738060_s_at | HIST2H4B | 2.1283581 | 0.000768843 |
| 11738131_a_at | RNF212 | 2.12696 | 0.000194818 |
| 11758202_s_at | ATPIF1 | 2.1260424 | 0.00042967 |
| 11736250_a_at | KIAA0101 | -2.126025 | 2.94447E-06 |
| 11728825_at | TRNP1 | 2.1253748 | 0.000319971 |
| 11725760_at | MEF2BNB | 2.1246502 | 0.000286496 |
| 11755091_a_at | ABCA7 | 2.123525 | 0.000401512 |
| 11716450_s_at | RFTN1 | -2.1233582 | 0.000286986 |
| 11753126_s_at | GJC1 | -2.122684 | 6.81266E-05 |
| 11725810_at | GUCA1B | 2.1220868 | 0.000247543 |
| 11743916_a_at | NPC1 | -2.1218824 | 0.000395146 |
| 11718986_a_at | IFI6 | 2.1211104 | 6.30155E-05 |
| 11726004_a_at | LAYN | 2.120104 | 4.28902E-05 |
| 11759525_at | GADD45B | -2.1197834 | 0.000295367 |
| 11724818_a_at | SH3KBP1 | -2.1180782 | 0.000304205 |
| 11717903_s_at | IER3IP1 | 2.1172593 | 8.76086E-06 |
| 11733269_at | NAT8L | -2.1171913 | 0.000299541 |
| 11736458_x_at | KCNK6 | 2.1163082 | 7.64977E-05 |
| 11729242_at | FBN2 | 2.1155605 | 0.000188962 |
| 11749843_a_at | BRMS1L | 2.1153605 | 0.000679209 |
| 11719912_a_at | KDM1B | -2.114546 | 0.001458752 |
| 11721956_at | RFX7 | -2.1132472 | 0.001043953 |
| 11729821_at | IL27RA | 2.1128786 | 7.61688E-05 |
| 11725584_at | HAS3 | -2.1111479 | 0.000558748 |
| 11761738_x_at | SNORD61 | -2.1110942 | 2.35824E-05 |
| 11761738_x_at | RBMX | -2.1110942 | 2.35824E-05 |
| 11728013_s_at | RIMKLB | -2.1094937 | 0.001486276 |
| 11722758_at | NPR3 | 2.1094074 | 0.000125708 |
| 11723950_a_at | PBK | -2.1079655 | 0.00010756 |
| 11757688_a_at | ANKHD1 | -2.1071196 | 0.008362245 |
| 11757688_a_at | ANKHD1-EIF4EBP3 | -2.1071196 | 0.008362245 |
| 11758838_at | SPTLC2 | 2.1069498 | 5.89095E-05 |
| 11752656_a_at | CBS | -2.1067796 | 2.99305E-05 |
| 11726633_s_at | TRIM8 | 2.1063242 | 0.000116598 |
| 11725718_a_at | CDC42 | -2.1053803 | 0.000710218 |
| 11755953_a_at | DMRTA1 | -2.1027846 | 0.000502493 |
| 11737955_a_at | C15orf40 | 2.1015937 | 6.02237E-05 |
| 11728206_a_at | FAM120A | -2.1008413 | 0.000686667 |
| 11749988_x_at | AUH | 2.1003616 | 4.35976E-06 |
| 11716823_s_at | BDH2 | 2.10022 | 0.001476234 |
| 11736694_x_at | TRIM38 | 2.099765 | 0.00015501 |
| 11724215_at | SPRYD4 | 2.098676 | 0.00063114 |
| 11725232_at | ATP8B2 | 2.0983286 | 1.53002E-05 |
| 11722409_at | GALNT7 | 2.0980697 | 0.00081579 |
| 11732515_x_at | BAK1 | 2.0973775 | 1.65756E-05 |
| 11754109_s_at | BIRC5 | -2.0971665 | 2.93623E-05 |
| 11737174_a_at | HMMR | -2.0969856 | 4.10439E-05 |
| 11716832_s_at | TM7SF3 | 2.095948 | 7.66816E-05 |
| 11742772_a_at | LSM14A | -2.0953362 | 2.99615E-05 |
| 11720280_at | KCNMA1 | -2.0951583 | 1.9391E-05 |
| 11723424_at | IFNAR1 | 2.0946417 | 0.000450953 |
| 11743076_a_at | PRKRA | -2.0941808 | 0.000103676 |
| 11730195_at | SEC14L2 | -2.0939991 | 0.000392691 |
| 11731641_s_at | TRAPPC10 | 2.0935407 | 3.98997E-05 |
| 11725788_a_at | CENPN | -2.0933063 | 1.51875E-05 |
| 11750598_s_at | TPX2 | -2.0932791 | 1.12706E-05 |
| 11722943_a_at | ATXN1 | 2.0926528 | 0.000146701 |
| 11729052_at | PPFIBP1 | -2.092135 | 0.002853518 |
| 11727458_at | KIF18A | -2.0917623 | 4.21246E-05 |
| 11727477_at | SAP30 | -2.0916317 | 0.003305205 |
| 11751219_x_at | GOLGA4 | -2.0915449 | 0.000287578 |
| 11746270_a_at | SLC19A1 | -2.0912554 | 0.002719247 |
| 11715898_a_at | TNIP1 | -2.0905595 | 0.00035119 |
| 11744468_at | SYNCRIP | -2.0892467 | 0.000263145 |
| 11723435_s_at | TTK | -2.0891051 | 0.000262581 |
| 11715604_x_at | LOC100505813 | 2.0887387 | 8.58519E-05 |
| 11715604_x_at | SPARC | 2.0887387 | 8.58519E-05 |
| 11723192_a_at | UHRF1 | -2.0882146 | 1.05605E-05 |
| 11720154_at | NRIP1 | -2.0881686 | 0.000608628 |
| 11740611_x_at | ZNF121 | -2.0881462 | 0.000802633 |
| 11717568_s_at | NQO1 | 2.0876918 | 0.000331871 |
| 11717440_a_at | EPS8 | -2.0861902 | 0.000908047 |
| 11721239_x_at | ATP5L | 2.0852232 | 2.09869E-05 |
| 11715788_a_at | SLC40A1 | 2.0842059 | 0.000518229 |
| 11735257_a_at | IL7 | 2.0838168 | 0.000839196 |
| 11761064_x_at | SPDYE2L | 2.082243 | 7.1873E-05 |
| 11725980_at | CFD | -2.0819502 | 7.79138E-05 |
| 11720319_at | SPAG5 | -2.0818362 | 0.000480042 |
| 11727610_at | ENSA | -2.0803123 | 0.003085593 |
| 11756354_a_at | RRP8 | -2.079118 | 0.001322121 |
| 11748136_a_at | MAD2L1 | -2.0777345 | 3.76393E-05 |
| 11758873_a_at | HPSE | 2.076669 | 0.009307426 |
| 11734657_s_at | SLC2A3 | -2.076587 | 0.000239108 |
| 11734657_s_at | SLC2A14 | -2.076587 | 0.000239108 |
| 11730834_a_at | KATNA1 | -2.0761447 | 7.4293E-05 |
| 11718578_a_at | GSTM3 | 2.0743368 | 0.000494235 |
| 11722815_at | RANBP2 | 2.0733104 | 0.001942697 |
| 11751123_a_at | CDC45 | -2.0701294 | 0.00089645 |
| 11753856_a_at | CKS2 | -2.0691013 | 0.000136236 |
| 11746575_a_at | LTBP3 | 2.068816 | 4.32323E-05 |
| 11716715_a_at | CTSB | 2.068463 | 8.31684E-05 |
| 11730507_at | NR0B1 | -2.0683892 | 0.000507597 |
| 11741554_x_at | CASP8 | 2.0683117 | 0.000328377 |
| 11716087_s_at | AKT1 | 2.0652328 | 0.000105549 |
| 11725441_a_at | FOXM1 | -2.0650616 | 5.39083E-05 |
| 11729920_at | C14orf45 | -2.0641143 | 0.001796833 |
| 11738999_a_at | SLC38A2 | -2.063966 | 0.000602958 |
| 11718828_s_at | ITM2C | 2.0627873 | 6.97776E-05 |
| 11728976_at | ZNF792 | 2.0616853 | 1.28499E-05 |
| 11754102_a_at | TMED3 | -2.0612793 | 0.000126513 |
| 11739512_a_at | TIMM50 | -2.061081 | 3.26479E-06 |
| 11729079_s_at | ESRRG | 2.0609047 | 0.000340772 |
| 11755062_a_at | KDELC1 | 2.0599656 | 0.000846135 |
| 11753258_s_at | EGFL8 | 2.0595717 | 8.23251E-05 |
| 11753258_s_at | PPT2 | 2.0595717 | 8.23251E-05 |
| 11753258_s_at | PPT2-EGFL8 | 2.0595717 | 8.23251E-05 |
| 11733352_a_at | NF2 | -2.0594409 | 0.000108027 |
| 11725425_s_at | RAB27A | 2.0580978 | 4.56463E-07 |
| 11754422_a_at | CARS2 | -2.0567667 | 0.00049669 |
| 11759602_a_at | C1orf63 | 2.055903 | 0.000422486 |
| 11733035_at | C7orf73 | 2.0549612 | 2.68988E-06 |
| 11760500_x_at | ZNF451 | 2.0546162 | 0.001563593 |
| 11722183_s_at | ATP6V1C1 | 2.054417 | 0.000392557 |
| 11724635_at | YOD1 | 2.0542762 | 0.001213206 |
| 11739385_x_at | ROCK2 | -2.0540864 | 0.001316712 |
| 11739365_x_at | FAM92A1 | -2.0510948 | 4.39522E-05 |
| 11742863_a_at | CD200 | -2.050448 | 0.000392002 |
| 11726409_a_at | C17orf39 | -2.0486674 | 5.47992E-05 |
| 11732032_a_at | HMGA2 | -2.0478673 | 0.000488849 |
| 11725575_a_at | EML6 | 2.0467777 | 0.00317483 |
| 11716377_s_at | GRN | 2.0467427 | 6.63802E-06 |
| 11744085_at | FAM100B | -2.0466905 | 0.000256765 |
| 11722236_at | FBXO2 | 2.046286 | 8.67262E-06 |
| 11747713_a_at | PRC1 | -2.04605 | 0.000167118 |
| 11726433_a_at | OSBPL3 | -2.044971 | 0.00031688 |
| 11724510_at | PMAIP1 | 2.0444446 | 0.000119766 |
| 11759200_at | CNTN1 | 2.0435436 | 0.000700122 |
| 11721456_x_at | SERPINA1 | 2.042814 | 0.000302286 |
| 11731864_at | NKX2-2 | 2.0407872 | 0.000379135 |
| 11723494_a_at | RNF166 | -2.0390062 | 0.002886866 |
| 11716919_a_at | PDIA5 | 2.0388906 | 0.000110636 |
| 11730253_a_at | AHCTF1 | -2.03868 | 0.000609368 |
| 11739507_a_at | PBX3 | -2.0385194 | 0.000489565 |
| 11729258_at | MNS1 | -2.0384915 | 3.7557E-05 |
| 11731694_s_at | HIST1H2AB | 2.0384166 | 0.000284506 |
| 11731694_s_at | HIST1H2AE | 2.0384166 | 0.000284506 |
| 11722244_at | TMEM27 | -2.0380332 | 0.000999145 |
| 11717710_a_at | MTFP1 | -2.0376453 | 0.000171514 |
| 11739973_s_at | NUAK1 | -2.037245 | 0.000141386 |
| 11724794_a_at | PGS1 | 2.036087 | 0.000333929 |
| 11736240_a_at | AAED1 | -2.0348244 | 7.08553E-05 |
| 11745035_x_at | LOC400879 | 2.0335915 | 0.000166879 |
| 11745035_x_at | FLJ39632 | 2.0335915 | 0.000166879 |
| 11745035_x_at | LOC100506303 | 2.0335915 | 0.000166879 |
| 11744762_a_at | FAM53A | 2.0317655 | 4.73604E-05 |
| 11763332_a_at | VPS36 | 2.0311234 | 0.001098763 |
| 11717381_a_at | FOPNL | -2.0308428 | 1.95986E-05 |
| 11759156_at | HIST1H2BI | 2.0307758 | 0.000982773 |
| 11759156_at | HIST1H2BC | 2.0307758 | 0.000982773 |
| 11759156_at | HIST1H2BE | 2.0307758 | 0.000982773 |
| 11759156_at | HIST1H2BF | 2.0307758 | 0.000982773 |
| 11759156_at | HIST1H2BG | 2.0307758 | 0.000982773 |
| 11744940_s_at | FAM108A1 | -2.029663 | 9.33618E-05 |
| 11744940_s_at | FAM108A4P | -2.029663 | 9.33618E-05 |
| 11758118_s_at | YARS | -2.0289838 | 0.000271464 |
| 11718159_at | NMI | 2.0283709 | 0.000716416 |
| 11718552_at | OTUD4 | -2.0259705 | 0.002391826 |
| 11754942_x_at | C17orf62 | 2.0250332 | 0.000103673 |
| 11754398_at | CXorf69 | 2.024993 | 0.005337512 |
| 11719004_at | ANKH | -2.0248454 | 5.33527E-05 |
| 11734043_at | SOGA3 | 2.0241413 | 0.000329845 |
| 11734043_at | KIAA0408 | 2.0241413 | 0.000329845 |
| 11758936_at | HNRNPR | -2.0229442 | 0.004171679 |
| 11729166_a_at | DCTD | -2.0226111 | 1.45188E-06 |
| 11715939_a_at | KHDRBS1 | -2.022582 | 7.16186E-06 |
| 11720523_at | RRP9 | -2.0215032 | 1.63238E-05 |
| 11729003_at | ADRB1 | 2.0209665 | 0.002521699 |
| 11715480_x_at | GABARAP | 2.0208 | 2.64961E-07 |
| 11726027_x_at | STAG3L4 | -2.0193071 | 0.000111801 |
| 11748791_a_at | AP5M1 | 2.0180078 | 2.38166E-06 |
| 11726708_a_at | FAM195B | -2.0173018 | 0.000298667 |
| 11718824_at | IGIP | 2.0165858 | 0.000966333 |
| 11758995_at | LOX | -2.016494 | 0.001631575 |
| 11720703_at | MYL4 | 2.016439 | 6.6938E-05 |
| 11759677_at | HERC4 | -2.0159078 | 0.001467315 |
| 11718943_a_at | AURKA | -2.0154269 | 0.000106626 |
| 11716315_at | MRPL15 | -2.0140378 | 2.85172E-05 |
| 11738592_at | BLID | -2.0139642 | 0.000569592 |
| 11731855_at | C10orf107 | 2.013906 | 0.001688775 |
| 11744465_x_at | PPP1R15A | -2.0130873 | 0.005793499 |
| 11720821_x_at | FUNDC2 | 2.011296 | 3.33088E-05 |
| 11743502_a_at | GTF3C2 | 2.0112445 | 6.42875E-05 |
| 11747278_a_at | MDH2 | -2.0102463 | 6.97083E-06 |
| 11730820_at | PCDHB9 | 2.0098562 | 0.000506662 |
| 11730820_at | PCDHB10 | 2.0098562 | 0.000506662 |
| 11731208_at | PTPRH | -2.0089808 | 0.000142183 |
| 11746157_a_at | ALKBH6 | 2.0086753 | 7.82266E-05 |
| 11724413_s_at | PPARA | -2.0085254 | 5.10402E-05 |
| 11747230_a_at | BUB1 | -2.008281 | 0.000366678 |
| 11722479_a_at | CUL5 | -2.008253 | 0.001336219 |
| 11741558_a_at | HOXB6 | 2.0067043 | 0.00026193 |
| 11745820_s_at | PLAGL1 | 2.005614 | 2.05763E-05 |
| 11754803_s_at | UCHL1 | 2.003719 | 2.68501E-05 |
| 11733906_a_at | THAP1 | -2.002781 | 3.63039E-05 |
| 11744636_a_at | OPRL1 | 2.0021896 | 0.000698181 |
| 11720758_s_at | IVNS1ABP | -2.0020497 | 0.000134416 |
| 11726329_x_at | GBP1 | 2.001461 | 0.000163988 |
| 11723864_s_at | MTMR11 | 2.0010772 | 0.001671483 |
| 11722162_a_at | PLK2 | -2.0006115 | 0.001266775 |
| 11732737_at | NUP62CL | 2.0003664 | 0.000157677 |
| 11715607_at | PPIB | -2.0001032 | 2.51079E-05 |
